# Enzymatic Synthesis and Molecular Modelling Studies of Rhamnose Esters Using Lipase from *Pseudomonas stutzeri*

**DOI:** 10.3390/ijms23042239

**Published:** 2022-02-17

**Authors:** Cecilia García-Oliva, Almudena Perona, Ángel Rumbero, Pilar Hoyos, María J. Hernáiz

**Affiliations:** 1 Biotransformation Group, Department of Chemistry in Pharmaceutical Sciences, Faculty of Pharmacy, Complutense University of Madrid, Plaza Ramón y Cajal, 28040 Madrid, Spain; ceciliag@ucm.es (C.G.-O.); alperona@ucm.es (A.P.); phoyosvi@ucm.es (P.H.); 2 Department of Organic Chemistry, Autonomous University of Madrid, Cantoblanco, 28049 Madrid, Spain; angel.rumbero@uam.es

**Keywords:** rhamnose esters, *Pseudomonas stutzeri* lipase, sugar fatty acid esters, enzymatic synthesis

## Abstract

Rhamnolipids are becoming an important class of glycolipid biosurfactants. Herein, we describe for the first time the enzymatic synthesis of rhamnose fatty acid esters by the transesterification of rhamnose with fatty acid vinyl esters, using lipase from *Pseudomonas stutzeri* as a biocatalyst. The use of this lipase allows excellent catalytic activity in the synthesis of 4-*O*-acylrhamnose (99% conversion and full regioselectivity) after 3 h of reaction using tetrahydrofuran (THF) as the reaction media and an excess of vinyl laurate as the acyl donor. The role of reaction conditions, such as temperature, the substrates molar ratio, organic reaction medium and acyl donor chain-length, was studied. Optimum conditions were found using 35 °C, a molar ratio of 1:3 (rhamnose:acyldonor), solvents with a low logP value, and fatty acids with chain lengths from C4 to C18 as acyl donors. In hydrophilic solvents such as THF and acetone, conversions of up to 99–92% were achieved after 3 h of reaction. In a more sustainable solvent such as 2-methyl-THF (2-MeTHF), high conversions were also obtained (86%). Short and medium chain acyl donors (C4–C10) allowed maximum conversions after 3 h, and long chain acyl donors (C12–C18) required longer reactions (5 h) to get 99% conversions. Furthermore, scaled up reactions are feasible without losing catalytic action and regioselectivity. In order to explain enzyme regioselectivity and its ability to accommodate ester chains of different lengths, homology modelling, docking studies and molecular dynamic simulations were performed to explain the behaviour observed.

## 1. Introduction

Lipases are among the most versatile biocatalysts; therefore, they find multiple applications at an industrial scale [1,2,3]. In particular, lipases have been widely used in the enzymatic synthesis of carbohydrate fatty acid esters [4,5,6,7,8], which constitute a very interesting group of non-ionic biosurfactants with important applications in the food industry [9]. For this purpose, they have shown some advantages over conventional surfactants, such as, biodegradability, non-toxicity, taste free and odour free, as well as being an environmentally benign alternative to petrochemical-derived surfactants [10].

Lipase-catalysed synthesis of sugar esters can be achieved by direct esterification of sugars with fatty acids or by transesterification with fatty acid vinyl esters [11]. So far, Novozym 435 from Novozyme (*Candida antarctica* lipase B immobilized on acrylic resin) has been the most popular enzyme used for this application. The use of the vinyl esters improves the production yield by driving the reaction through the tautomerization of the enol product. These synthetic reactions are preferentially carried out in non-aqueous media. However, the enzymatic methods often suffer from slow reaction rates and low production yields, which are mainly caused by the poor solubility of carbohydrates in most conventional organic solvents. Sugars are only soluble in a few polar solvents, such as pyridine, dimethyl sulfoxide (DMSO), and dimethyl formamide (DMF). However, these solvents have been found to be serious deactivators of enzymes and not the optimal choice in some industrial applications. Therefore, the selection of appropriate solvents is crucial to ensure good yields in the enzymatic synthesis of sugar esters [11].

Lipase from *Pseudomonas stutzeri* (PSL) has proven to be a versatile biocatalyst, accepting a broad range of substrates, such as wood sterols [12], benzoins [13], lauric acid [14] and alcohols [15], and being active in hydrophobic solvents, such as toluene [15], and in hydrophilic solvents such as acetone, 1,4 dioxane and THF [14,16,17]. The enzyme displayed activity also in other solvents such as cyclopentylmethylether (CPME) and 2-MeTHF [18]. However, there are very few examples where PSL is used as a biocatalyst in the synthesis of carbohydrate fatty acid esters. Only Bernal et al. have described the enzymatic synthesis of lactulose palmitate with a very low yield (5%) and moderate yield (20–30%), using soluble PSL or PSL immobilised on octyl-glyoxyl silica, respectively, using acetone as the reaction media [19,20]. On the other hand, this lipase has been successfully employed in the regioselective deacylation of peracetylated monosaccharides [21].

Rhamnolipids (RLs) are one of the most studied biosurfactants due to its remarkable surface activity, emulsifying properties [22,23] and its numerous applications in cosmetics, detergents and bioremediation [24,25,26]. Besides, they also display antimicrobial, anticancer and immunomodulation properties which made them also useful in pharmaceutical, food and health-care industries [27]. RLs are mainly produced from *Pseudomonas aeruginosa*. However, the bulk application of RLs in industry is not feasible due to the high production costs [28,29], attributed to low-efficiency, and, therefore, infeasible, industrial fermentation. Specific barriers to the industrial production of RLs include excess foaming, comparatively expensive raw material and costly downstream processing [30,31,32]. Thus, lipase-catalysed synthesis of RLs could facilitate the industrial production of rhamnolipids.

In this work we describe for the first time that rhamnose fatty acid esters can be obtained directly from rhamnose and fatty acids (or their corresponding vinyl esters) employing PSL as biocatalyst. The reaction conditions have been studied, including reaction temperature, solvent, ratio sugar/acyl donor and fatty acid chain length. The optimised parameters were applied to enzymatic synthesis of rhamnose esters obtaining high yields. Molecular modelling studies were carried out to in order to explain the binding mode of rhamnose to PSL and the high yields obtained.

## 2. Results and Discussion

### 2.1. Enzymatic PSL Synthesis of 4-O-lauroylrhamnose

**Scheme 1:**
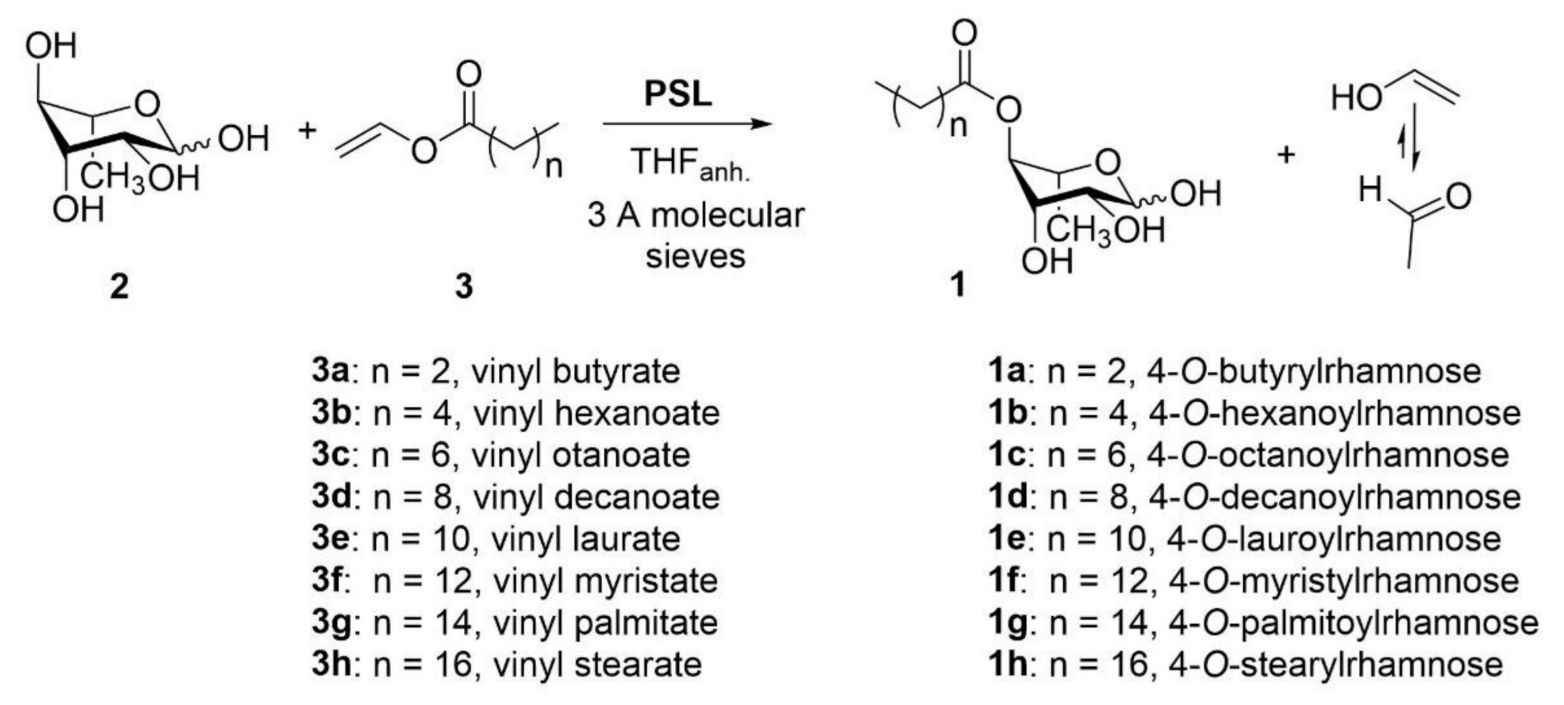
Lipase-catalysed synthesis of **1** using L-rhamnose and fatty acid vinyl esters as substrates.

With the aim of preparing a novel sugar ester of interest, PSL was evaluated in the synthesis of lauroylrhamnose (**1e**, Scheme 1) through a transesterification process, employing L-rhamnose (**2**) and vinyl laurate (**3e**) as the acyl donor (Scheme 1).

As this lipase had previously shown an excellent behaviour in the resolution of secondary alcohols through transesterification reactions performed in THF [16], we first studied the PSL-catalysed synthesis of **1e** in anhydrous THF at 35 °C (Scheme 1) with a molar ratio of rhamnose: acyl donor of 1:5. THF was selected because polar solvents favour the solubility of **2**, overcoming one of the main problems of the enzymatic synthesis of sugar fatty acid esters, the low solubility of sugars in the organic solvents commonly employed in lipase-catalysed reactions [5,9]. Thus, the use of PSL and THF was proposed as a suitable combination to initiate the study of this biocatalytic synthesis of **1e** at a 3 mL scale. Sugar ester synthesis was performed in the presence of an anhydrous solvent with the aim of favouring transesterification over hydrolysis, employing freshly distilled THF and adding 3 Å molecular sieves to the reaction medium. Additionally, the lipase and the substrates were also dried before their use in order to avoid the formation of the non-desired lauric acid.

In the Bradford assay, the protein content in the lipase commercial preparation was determined to be 19% and the lipase activity was found to be 44 IU. To test the capability of this lipase to mediate the transesterification of **2**, an initial 8.2 IU/mL concentration of PSL was employed.

**Figure 1:**
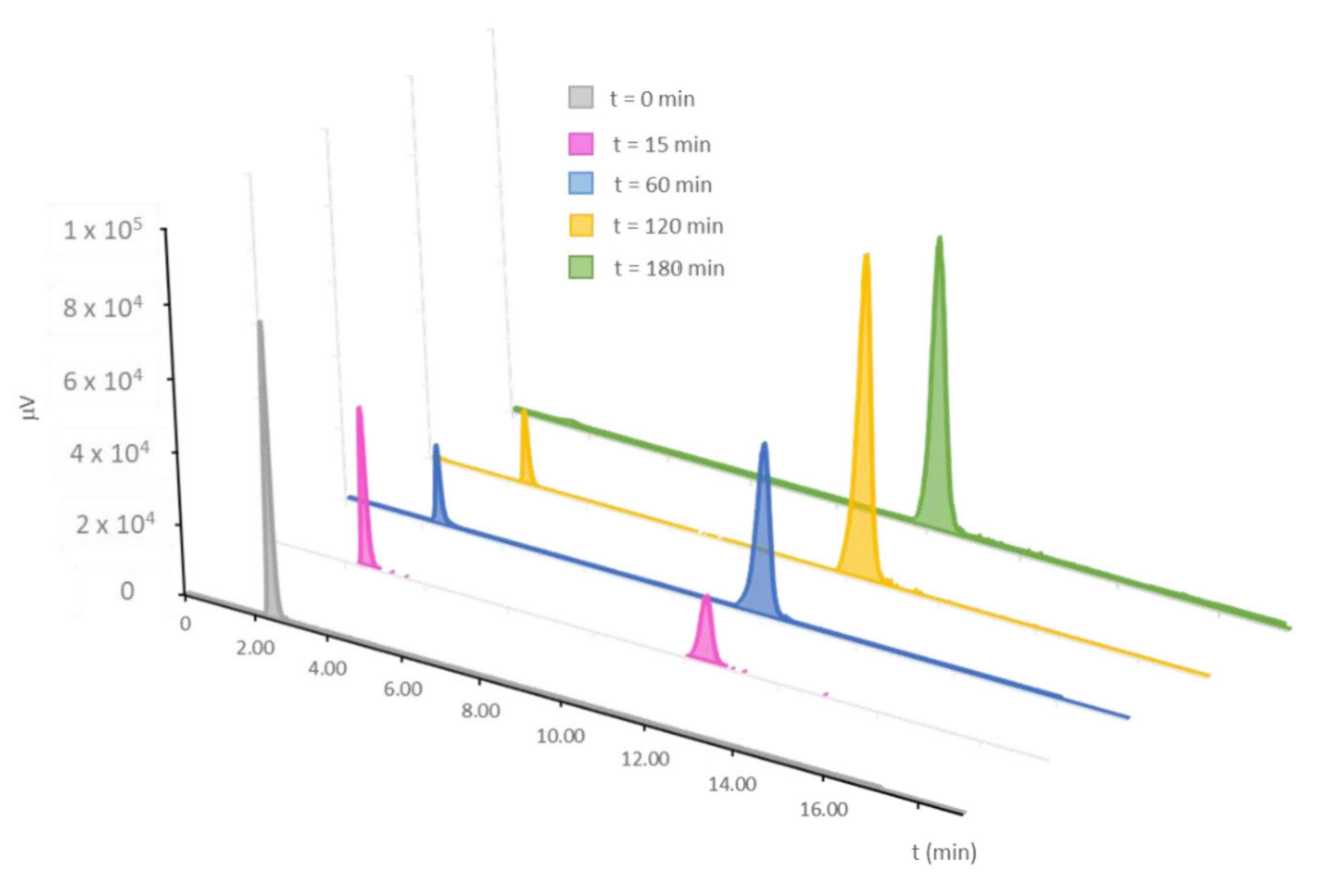
HPLC data at different times of the PSL-catalysed transesterification of **2** using **3e** as acyl donor in THF at 35 °C.

The enzymatic reaction progress was monitored by TLC and HPLC, detecting the rapid consumption of the substrate **2** and the corresponding formation of product **1e**. The chromatograms show the appearance of just one peak during the transesterification reaction (Figure S1 in Supplementary Materials), which indicates the formation of a single monoester, as it is described below. As can be seen in Figure 1, maximum conversion (99%) and a single product were reached in a very short reaction time (only after 3 h of reaction). The reaction was allowed to continue until 6 h, but no other products could be detected by HPLC.

**Figure 2:**
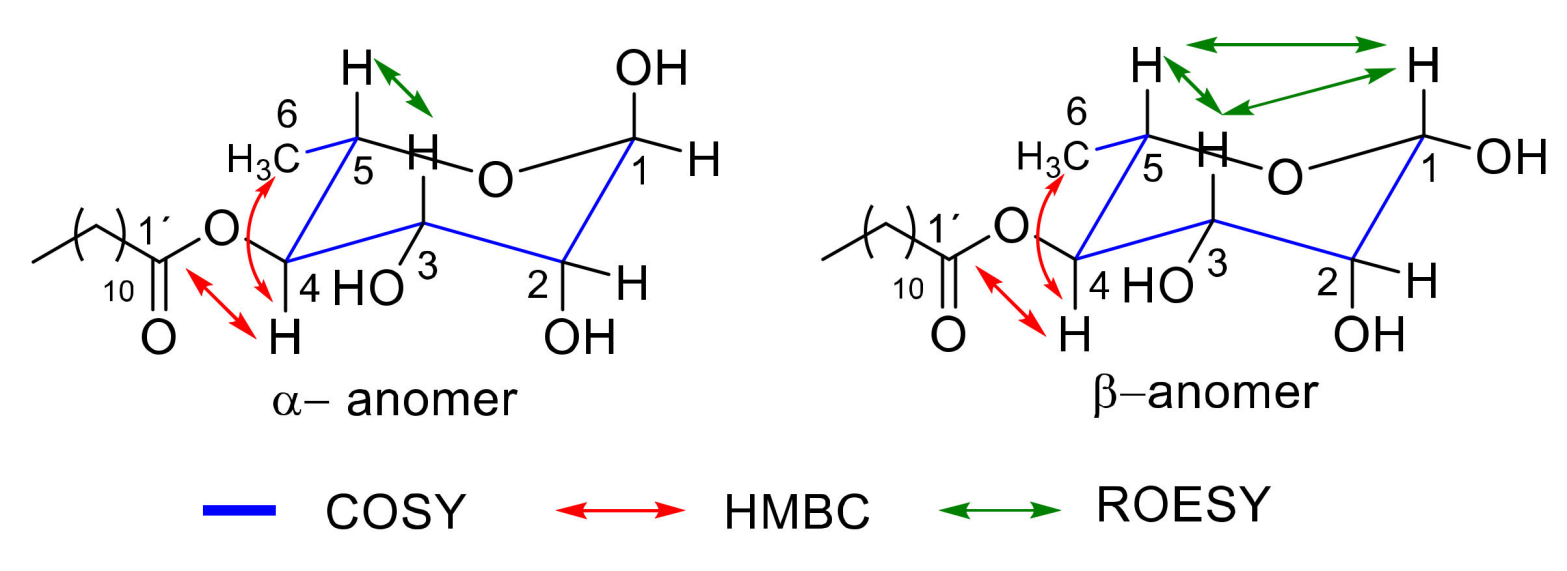
Most representative signals of 2D RMN (COSY, HMBC and ROESY) spectra of **1e**.

The reaction was then scaled up to 30 mL volume to facilitate the isolation of the product, achieving similar conversion results. After 3 h, maximum conversion was reached, and the product was purified by silica column chromatography and characterised by NMR (Figures S2–S7, Supplementary Materials) and MS (Figure S8, Supplementary Materials). MS analysis confirmed the existence of a single rhamnose monoester product with a molecular formula of C_18_H_34_O_6_ (*m/z* 369.2239 [M^+^Na]^+^). The NMR spectrum of compound **1e** revealed that PSL was highly selective towards the formation 4-*O*-lauroylrhamnose, and a mixture of α- and β- anomers was observed, which was exclusively found in the reaction medium. The relation of α/β was determined by the integration of signals corresponding to H-1/H-4 (α anomer, 57%) and H-1/H-4 (β anomer, 43%) on the ^1^H-NMR spectrum. Complete signal assignment was based from 1D NMR (^1^H, ^13^C and DEPT) and 2D RMN (COSY, HSQC, HMBC and ROESY) (Figures S4–S7 in Supplementary Materials). The position of the alkyl chain was confirmed by the HMBC spectrum, showing a correlation between H-4 and C-1’, which is indicated by the presence of the acyl group on C-4. Additionally, the relative configuration of C-1 was determined in the base of the ROESY spectrum, showing a correlation between H-1 and H-3/H-5 of the β-anomer (Figure 2 and Figures S4–S7 in Supplementary Materials). To the best of our knowledge, this is the best result described for the synthesis of rhamnose esters, not only because of the excellent conversions, but also for the high regioselectivity displayed by PSL and the short reaction time required to afford these results.

Compound **2** has been scarcely employed in the lipase-catalysed synthesis of sugar esters. Lipase B from *Candadia antarctica* (CAL-B) is the most frequently used enzyme for sugar ester synthesis in organic solvents [9]. Nott et al. described that CAL-B is able to recognise rhamnose as a substrate in the esterification reaction with myristic acid, affording four regioisomers of the rhamnose monoester [33]. On the other hand, our results are in agreement with those reported by Raku et al. [34], who demonstrated that 4-*O*-vinyl adipoyl-rhamnose monoesters were synthesised in a moderate yield (58% yield), using lipase from *Pseudomonas sp.* in pyridine.

Our excellent results led us to choose the enzymatic synthesis of **1e** as the standard reaction to optimise different parameters (temperature, organic solvent, the length of the vinyl ester chain).

### 2.2. Temperature Optimisation

**Figure 3:**
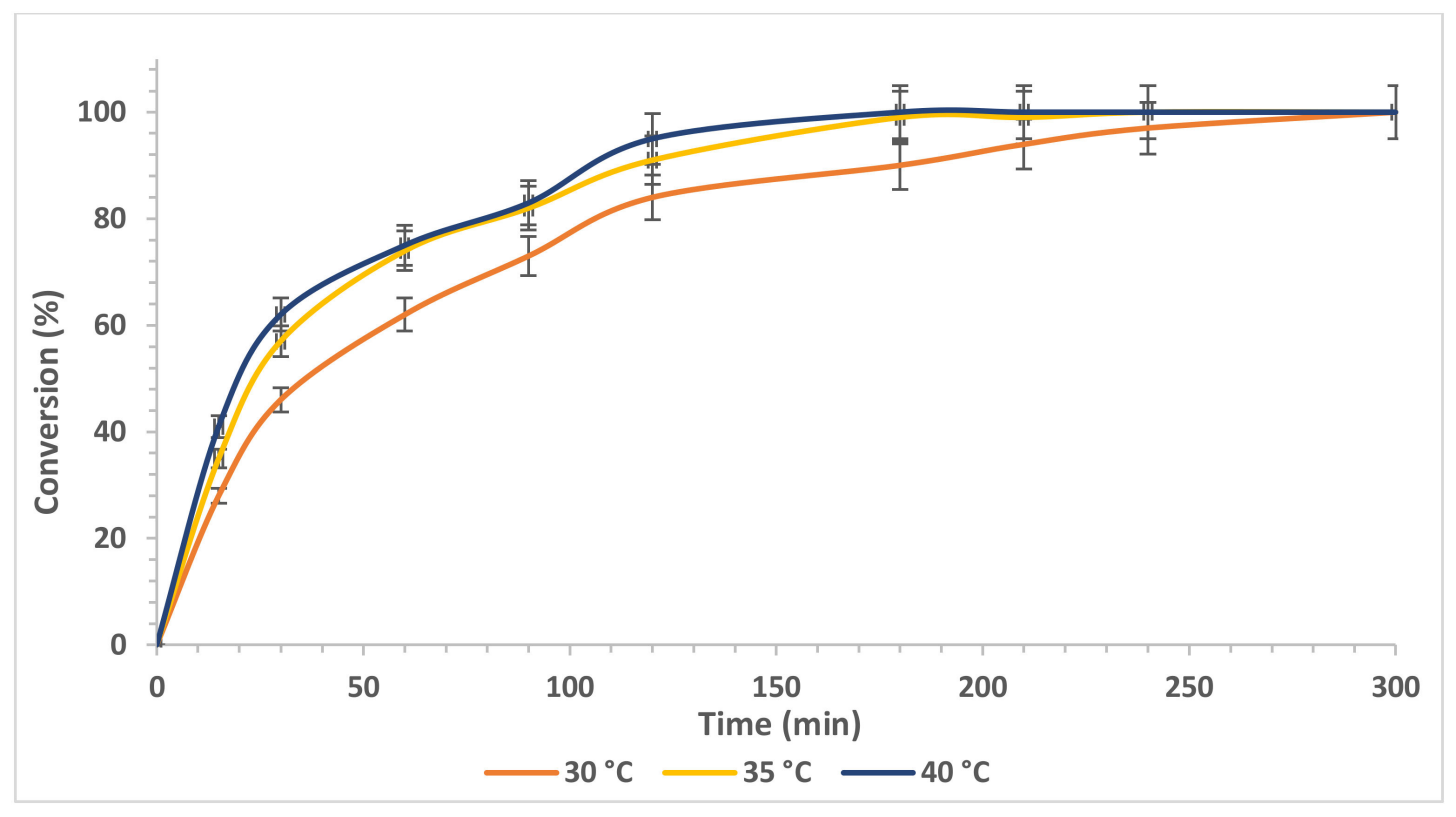
Synthesis of **1e** catalysed by PSL in anhydrous THF at different temperatures (30 °C, 35 °C and 40 °C).

In order to study the effect of temperature in the reaction time, transesterification of **2** and **3e** in THF catalysed by PSL was carried out at three temperatures (30 °C, 35 °C and 40 °C). As can be seen from Figure 3, in all cases the reactions proceeded similarly, with no signs of any lipase thermal deactivation under the reaction conditions. Working at 35 °C and 40 °C, transesterification increased rapidly with time, reaching 85% conversion after 2 h and a maximum of 99% after 3 h, slightly faster than the 4 h needed to reach the same conversion at 30 °C. Thus, as no significant differences were appreciated between the performance at 35 or 40 °C, 35 °C was fixed as the working temperature.

### 2.3. Effect of Substrates Molar Ratio

**Figure 4:**
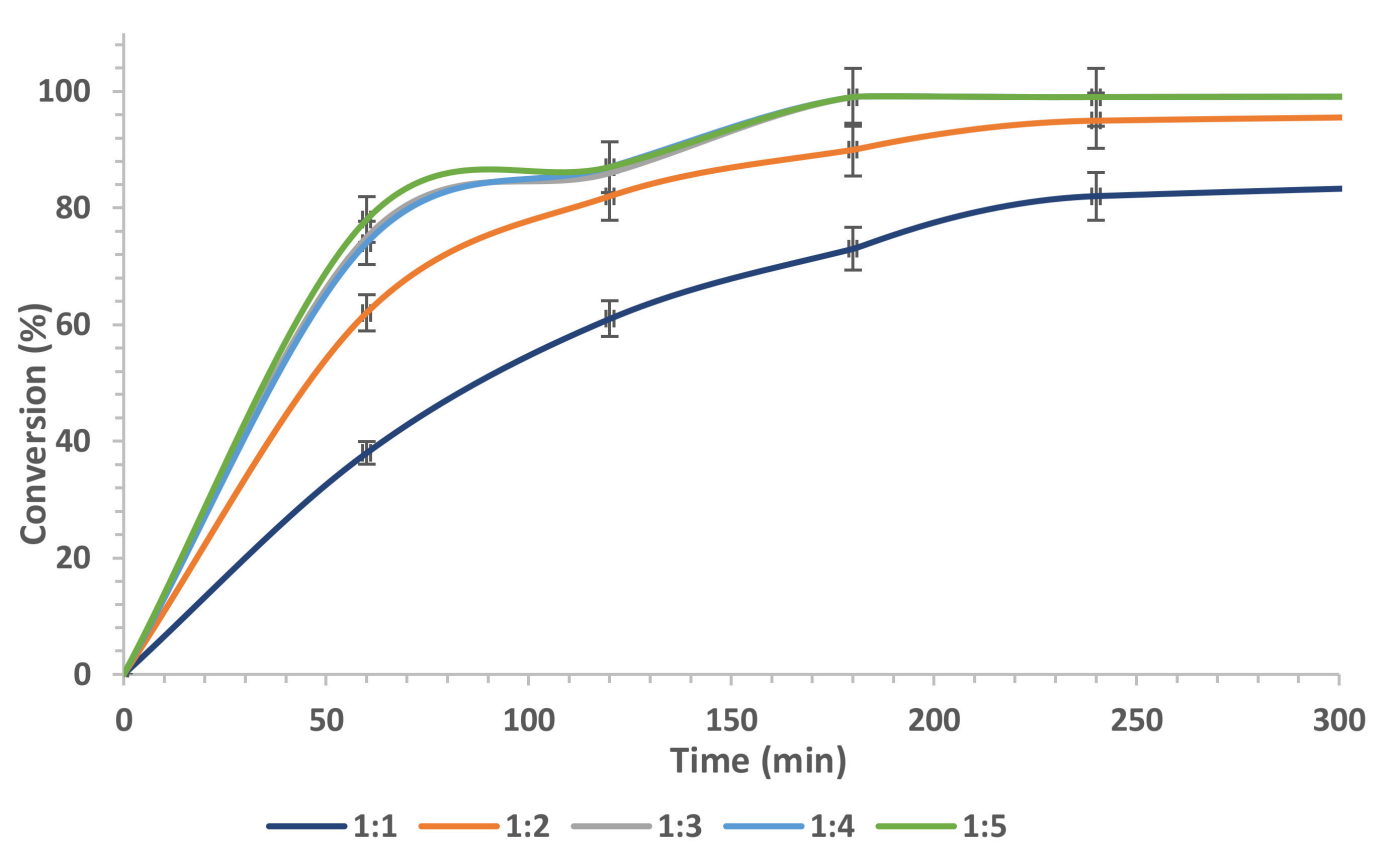
Influence of the substrate molar ratio (L-rhamnose: vinyl laurate) on the conversion.

Most enzyme-catalysed reactions are reversible. In order to shift the reaction in the desired direction, one of the substrates is often used in considerable excess [4]. On the other hand, increasing the amount of the acyl donor could aid in forming the acyl-enzyme intermediate and reduce the reaction time [35]. Therefore, different molar ratios (L-rhamnose: vinyl laurate), from 1:1 to 1:5 were investigated in the transesterification reaction mediated by PSL in anhydrous THF at 35 °C. As shown in Figure 4, the conversion yield was enhanced with the increasing molar ratio rhamnose: vinyl laurate up to 1:3: However, higher concentrations of the acylating agent did not significantly increase the conversion yield of **1e**. It is worth mentioning that no change in the regioselectivity of PSL occurred as the substrate molar ratio increased, indicating the rigorous regioselectivity of PSL toward **2**, and no rhamnose diesters were formed at higher acyl donor concentrations. Therefore, the optimal substrate molar ratio was stablished as 1:3.

### 2.4. Effect of the Organic Reaction Medium

One of the main problems in the enzymatic synthesis of sugar esters is the selection of a suitable solvent, one able to dissolve both substrates (sugar and fatty acid) [4]. The solubility of sugars and fatty acids in organic solvents are generally markedly different, thus complicating the esterification reaction since a high concentration of both the reactants within a single phase is difficult to achieve. Furthermore, the solvent must not adversely affect the stability and activity of the lipase.

The use of water-miscible solvents has the advantage that hydrophilic substrates (sugars) are solubilised to some extent without the addition of solubilising reagents to facilitate the esterification of the substrates [36,37]. In addition, water-miscible organic solvent systems can reduce the mass-transfer limitations, leading to faster reaction rates for hydrophobic compounds. However, a water-miscible solvent removes water from the lipase molecule, which is essential for catalytic activity; as a consequence, this water removal might promote the deactivation of the enzyme [38].

**Figure 5:**
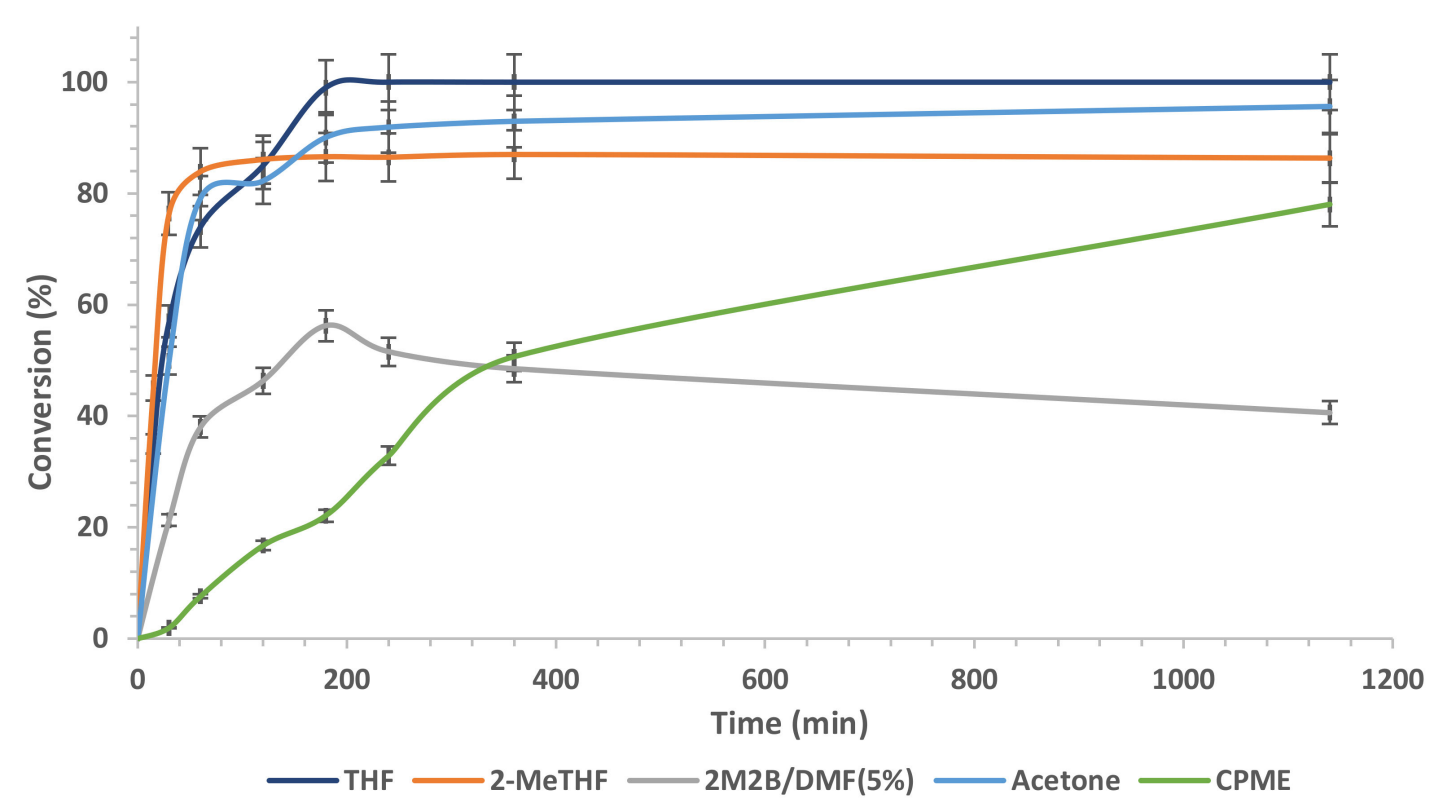
Comparison of conversion achieved with different organic solvents in the PSL-catalysed synthesis of **1e**.

PSL has been reported to be active and stable in hydrophobic solvents, such as toluene [15] and in hydrophilic solvents such as acetone, 1,4 dioxane, 2-MeTHF and THF [14,17]. As it was described above, synthesis of **1** was firstly proposed in THF, but it was also investigated in the presence of other pure polar solvents with the aim of favouring the solubility of **2**. 2-MeTHF, 2-methyl-2-butanol (2M2B)/DMF (5%), acetone and CPME were selected because polar solvents promote the solubility of the monosaccharide, which enables the development of this reaction at higher substrate concentration [5]. Reactions were carried out at an analytical scale, employing **3e** in a molar ratio 1:3 at 35 °C with 3 Å molecular sieves, and their progress was monitored by TLC and HPLC. In all cases, only the monoester **1e** was formed in the medium, maintaining the excellent regioselectivity of PSL, but not all the solvents gave the results afforded with THF. The comparison of the progress of the different reactions is shown in Figure 5. After 3 h of reaction, the best results for the synthesis of **1e** were reached in THF (99% conversion), acetone (92% conversion) and 2-MeTHF (86% conversion), and a considerably lower conversion was found with 2M2B/DMF and CPME (52% and 33%, respectively). 2M2B has been frequently used in the enzymatic preparation of sugar esters in combination with polar solvents such as DMF [39]. However, the presence of this hydrophilic solvent may interfere with the lipase behaviour and increase the moisture of the medium, preventing it from reaching conversions higher than 60%. On the other hand, the reaction rate was significantly slower when CPME was employed, requiring at least 20 h to reach 80% conversion. The monosaccharide was not completely soluble in this solvent, probably hampering the optimal action of the lipase. Compound **2** was solubilised as it was consumed in the transesterification reaction, which induced the deceleration of the process.

Therefore, the most suitable compromise between solubility and lipase activity was found with THF, which was then employed to prepare other fatty acid esters of **2**.

### 2.5. Influence of the Acyl Donor Chain-Length

To investigate the influence of the acyl donor chain-length, vinyl esters of fatty acids with chain lengths between C4 and C18 were used as substrates (Scheme 1). Reactions were performed at a semi-preparative scale, employing anhydrous THF at 35 °C, and their progress was monitored by TLC and HPLC. As described in the Experimental Section, for esters of butyric, hexanoic, octanoic, decanoic and lauric acids, acetonitrile:water 70:30 (*v/v*) was used as the mobile phase, for myristic acid it was acetonitrile:water 80:20 (*v/v*), and for palmitic and stearic acids it was 90:10 (*v/v*). A similar change in the solvent mixture, employed as the mobile phase as the length of the chain is increased, has already been described by Ferrer et al. [40] when carrying out an investigation on the regioselective synthesis of fatty acid esters of maltose, leucrose, maltotriose and n-dodecyl maltosides [40]. Zhao et al. also described similar experimental conditions when carrying out an investigation on enzymatic synthesis of glucose-based fatty acid esters [41].

As it is shown in Figure 6, all vinyl esters led to a considerably high conversion after 2 h of reaction. The products were purified by silica column chromatography and characterised by NMR and an accurate-mass analyses, which allowed one to differentiate the chain length (Figures S9–S15, Supplementary Materials).

Short and medium chain acyl donors (**3a**–**3e**) allowed maximum conversions after 3 h, and long chain acyl donors (**3f**–**3h**) required a longer reaction time (5 h) to get 99% conversions. The effect of the fatty acid chain over the reaction course depends on the lipase and the reaction conditions [4]. For example, Arniza et al. described a system with Lipozyme^®^ TL IM (*Thermomyces lanuginosus* lipase adsorbed onto silica gel) that displayed great selectivity towards a shorter fatty acid chain length in the preparation of sorbitol esters in *tert*-butanol [42]. On the contrary, Zhao et al. observed that the Lipozyme^®^ TL IM mediated transesterification of glucose in an ionic liquid IL/2M2B bisolvent system increased with the elongation of the chain length of the acyl donor. The same trend was observed with the Novozyme 435 catalysed transesterification of methyl glucoside in IL/2M2B [41].

After the complete consumption of **2**, the products **1a**–**1h** were purified and characterised, revealing again an excellent regioselectivity of PSL in the transesterification of **2**. In all cases, we have observed only monoacylation which occurred at the C-4 hydroxyl group, and no traces of diesters could be detected in the medium.

### 2.6. Homology Modelling, Docking Studies and Molecular Dynamic Simulation

#### 2.6.1. Docking of Rhamnose Anomers to PSL and Structure Stability Studies by Molecular Dynamic Simulation

In order to explain the excellent regioselectivity achieved by PSL in the transesterification reaction between rhamnose and vinyl esters, we carried out a molecular modelling study by homology of PSL and the already available model of *Pseudomonas aeruginosa* lipase (79.79% of identity with PSL).

**Figure 7:**
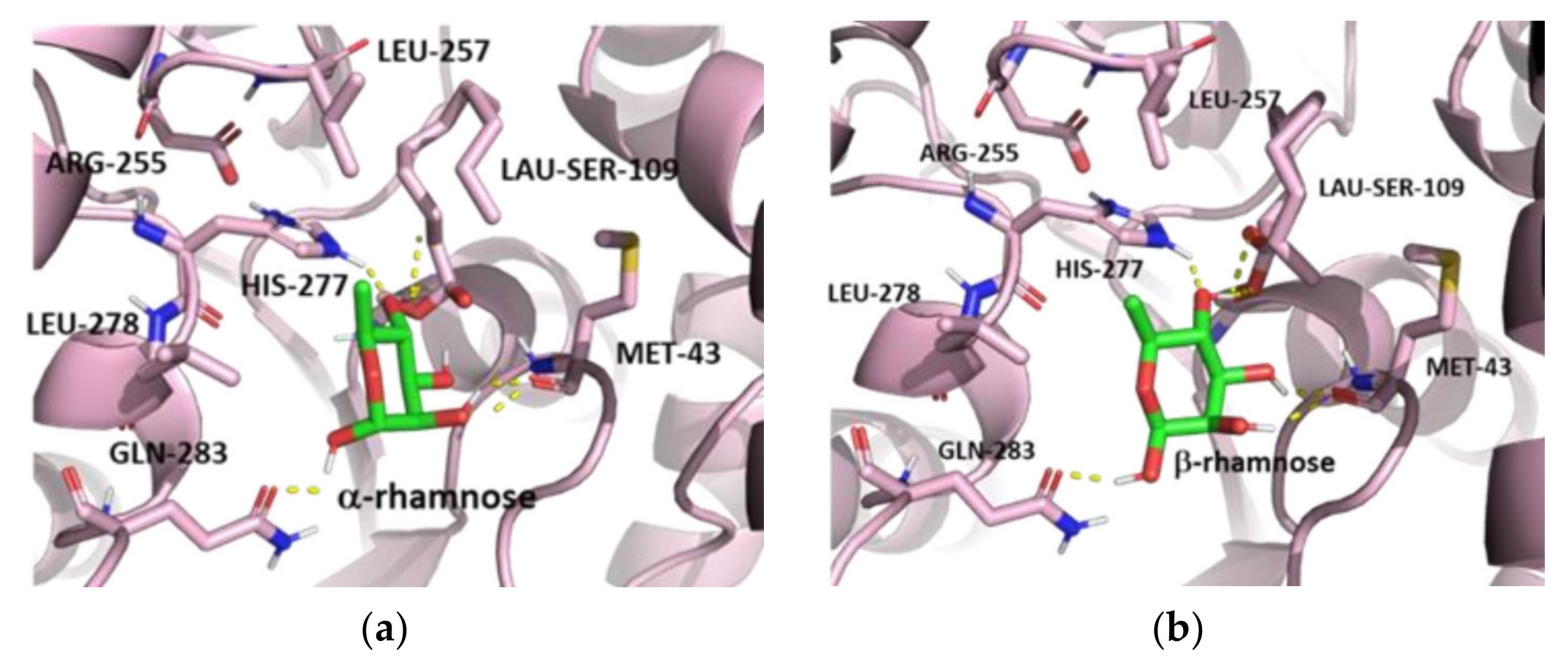
PSL active site with docked rhamnose: (**a**) α-rhamnose and (**b**) β-rhamnose. PSL is depicted in cartoon pink, principal residues of the enzyme are depicted in pink sticks and sugars in green sticks, hydrogen bond interactions are depicted as yellow dash lines.

With this aim, the active site of the enzyme was identified as the only cavity available in the structure with the catalytic triad residues His X, Asp Y and Ser Z (Figure 7). Visual inspection of the cavity indicated that other residues, commonly found in other lipases surrounding the catalytic triad, could be involved in the molecular recognition of the substrates. Bearing in mind the catalytic mechanism of the enzyme [43], we performed a molecular docking simulation of α- and β-rhamnose in the active site of PSL where the catalytic serine (SER-109) was already acetylated using its most favourable orientation pose, obtained in previous docking experiments between the PSL and the vinyl laurate chain.

We chose to place the docking box in the catalytic pocket, including all residues implicated in the enzyme mechanism. We used Autodock 4.2 [44] with reasonable flexibility of the ligand and protein. Among the several docking poses obtained from the interaction between the ligand and the target protein, the ones exhibiting the best docking score, which estimates the strength of the interaction, were chosen. Additionally, the distance between the residues involved in the transesterification reactions as HIS-277 and SER 109 were considered.

Figure 7 shows the binding modes adopted by the two rhamnose anomers in their interaction with the PSL model. The best score poses were the ones with the OH– group in position 4 of the hexose ring, which was closer to laurated-SER-109. The rhamnoses were anchored to the lipase cavity by multiple hydrogen bonds. In particular, α-rhamnose (Figure 7a) was stabilised in the active site by strong hydrogen bonding interactions with the catalytic residues SER-109 acylated, HIS-277 and with MET-43 and GLN-283, and distances that allowed the reaction (2.6 Å to HIS-277, and 2.5 Å and 2.8 Å to laurated-SER-109). Moreover, van der Waals interactions between the residues LEU-257, LEU-278 and the methyl group of the α-rhamnose set the pose. β-Rhamnose was also anchored in the catalytic pocket with the hydrogen bonds between hydroxyl groups of the sugar and the laurated-SER-109, HIS-277, MET-43 and GLN-283. Methyl group of the β-rhamnose interacted with LEU-257 and LEU-278. The distances between the hydroxyl group at C-4 of the hexose ring and HIS-277 (1.8 Å) and laurated-SER-109 (2.7 Å and 2.9 Å) are the best suited for the reaction (Figure 7b).

**Figure 8:**
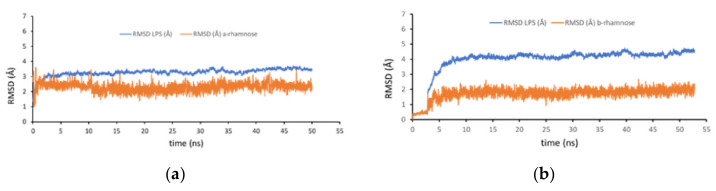
RMSD values relative to the starting structure along 50 ns of MD simulation of PSL complexed to (**a**) α-rhamnose and (**b**) β- rhamnose. The Y axis shows the RMSD values in Å and the X axis the time in nanoseconds. RMSD values are represented in blue for the lipase and orange for rhamnoses.

The structural stability of the α- and β-rhamnose poses as well as the bond distances between rhamnoses and the residues involved in the esterification reaction have been studied by 50 ns of a molecular dynamics simulation using THF as the solvent, performed with AMBER [45]. To assess convergence, the root-mean-square deviation (RMSD) values of the PSL complexes were calculated over the simulation time (Figure 8). The results show that the protein is stable in both dynamics at the beginning of the simulation, which corresponds to the equilibration stage of the dynamics, the protein accommodates its structure, reaching RMSD values of between 3–4 Å, with respect to the starting structure, as expected for model. Once the protein has been accommodated, it remains stable, without fluctuations in the RMSD values throughout the simulation. Regarding the rhamnoses, the α and β anomer, remained at their position along the simulation, no significant changes were observed in RMSD values with respect to the docked structure, reaching values smaller than 3 Å.

**Figure 9:**
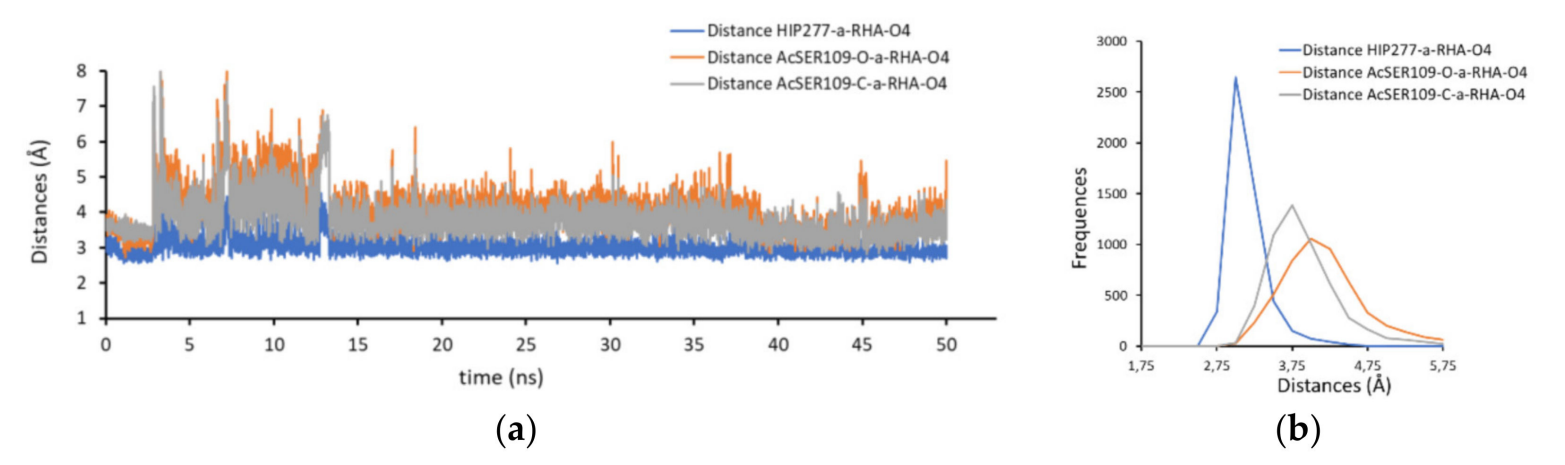
(**a**) Distance values relative to the starting structure along 50 ns of MD simulation of PSL complexed to α-rhamnose (**8**). The Y axis shows the distances values in Å and the X axis is the time in nanoseconds. (**b**) Distribution of the distance values along the simulation. In both charts, distance values between HIS-277 and C-4 hydroxyl group of α-rhamnose are represented in blue, between ester oxygen of laurated-SER-109 and C-4 hydroxyl group of α-rhamnose in orange, and between ester carbon of laurated-SER-109 and C-4 hydroxyl group in grey.

**Figure 10:**
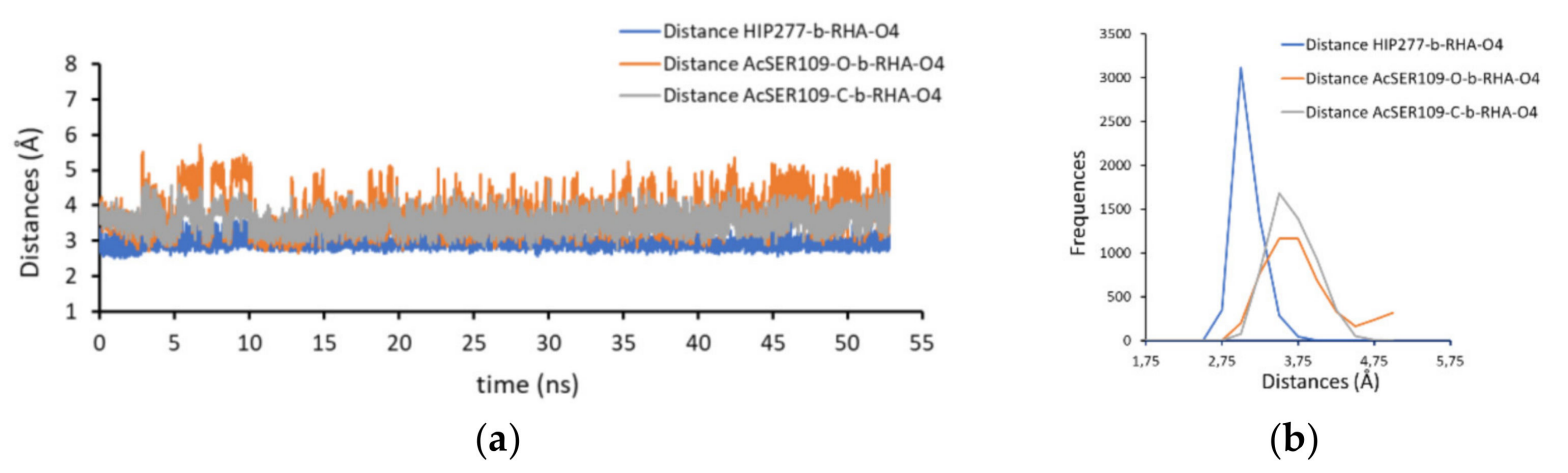
(**a**) Distance values relative to the starting structure along 50 ns of MD simulation of PSL complexed to β-rhamnose (**9**). The Y axis shows the distances values in Å and the X axis is the time in nanoseconds. (**b**) Distribution of the distance values along the simulation. In both charts, distance values between HIS-277 and C-4 hydroxyl group of α-rhamnose are represented in blue, between ester oxygen of laurated-SER-109 and C-4 hydroxyl group of α-rhamnose in orange, and between ester carbon of laurated-SER-109 and C-4 hydroxyl group in grey.

Finally, we have monitored the distances between the main atoms involved in the transesterification reaction with the aim to evaluate the selectivity of the reaction. After analysing the distance values along the two simulations, we have observed that the distance between HIS-277 and α-rhamnose remains stable along the simulation, with values smaller than 3 Å. The distance between the sugar´s C-4 hydroxyl group and the oxygens of the laurated-SER-109 fluctuated during the first 10 ns of the simulation, then values stabilised to distances that allowed the reaction, around 3,5 Å (Figure 9a,b). The behaviour of β-rhamnose along the simulation is similar to the α anomer, and after 10 ns the values were stable and allowed the reaction. It is noteworthy that β-rhamnose distance values did not reach the 5 Å (Figure 10a,b).

**Figure 11:**
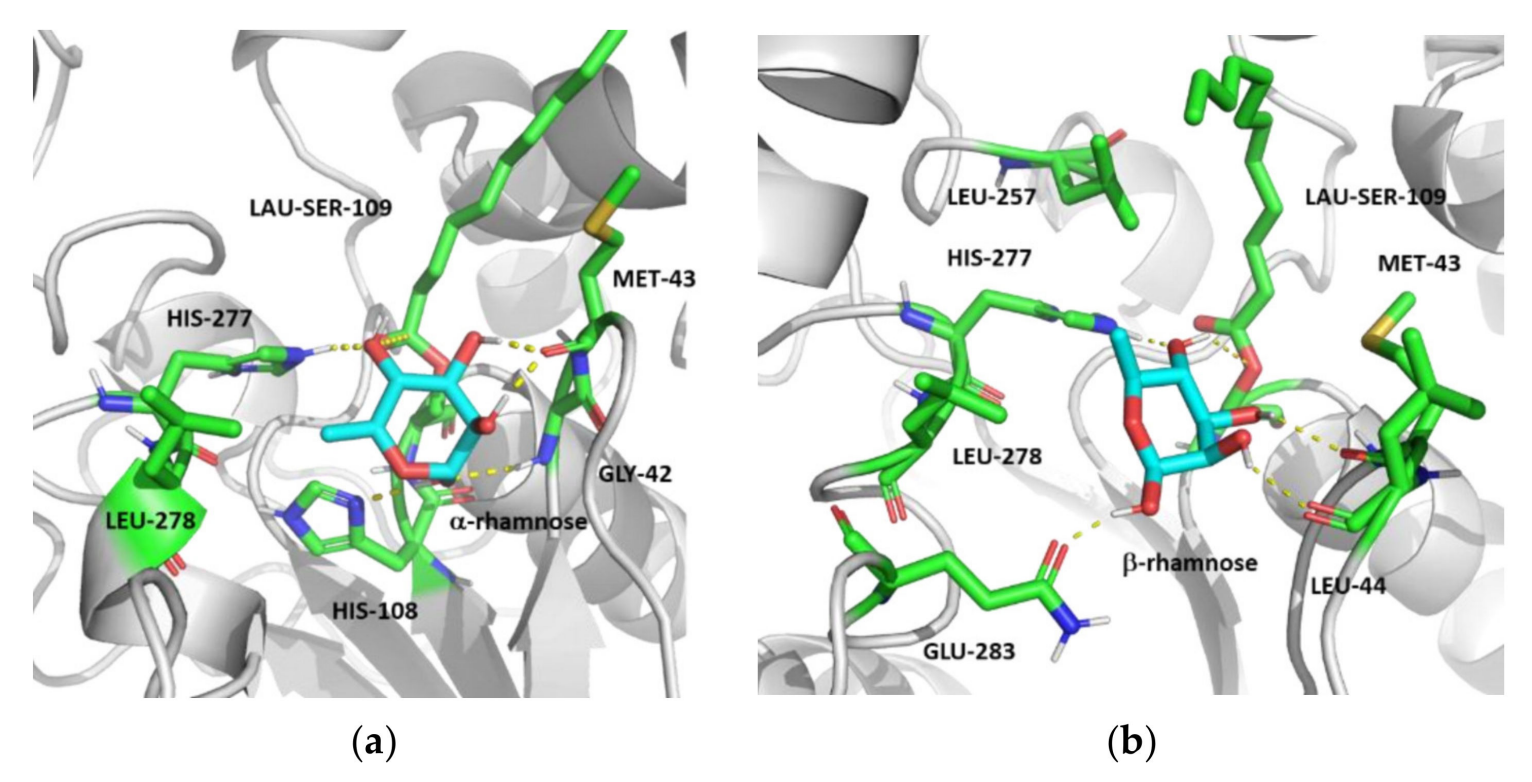
Three-dimensional view of the interaction after 50 ns of MD simulation between PSL and (**a**) α-rhamnose and (**b**) β-rhamnose. Enzyme is depicted in cartoon grey, principal residues of the enzyme are depicted in green sticks and sugars in cyan sticks.

Most of the predicted interaction observed in docking studies are validated with the simulation results; α-rhamnose is anchored at the active site by strong hydrogen bonding interactions, the C-4 hydroxyl group interacts with laurated-SER-109 and HIS-277, the C-3 and C-2 hydroxyl groups interact with MET-43, and the C-1 hydroxyl group with GLY-42 and HIS-108. The C-5 methyl group is stabilised by the van der Waals interaction with Leu-278 (Figure 11a). The position of β-rhamnose is fixed by hydrogen bond interactions between the C-4 hydroxyl group and HIS-277 and laurated-SER-109, the C-3 hydroxyl group interacts with MET-43, the C-4 hydroxyl group forms a hydrogen bond with LEU-44, and the C-1 hydroxyl group with GLU-283. Van der Waals interactions are observed between the C-5 methyl group LEU-278 and LEU-257 (Figure 11b).

#### 2.6.2. Docking of Vinyl Esters of Fatty Acids with Chain Lengths between C4 and C18 to PSL

**Figure 12:**
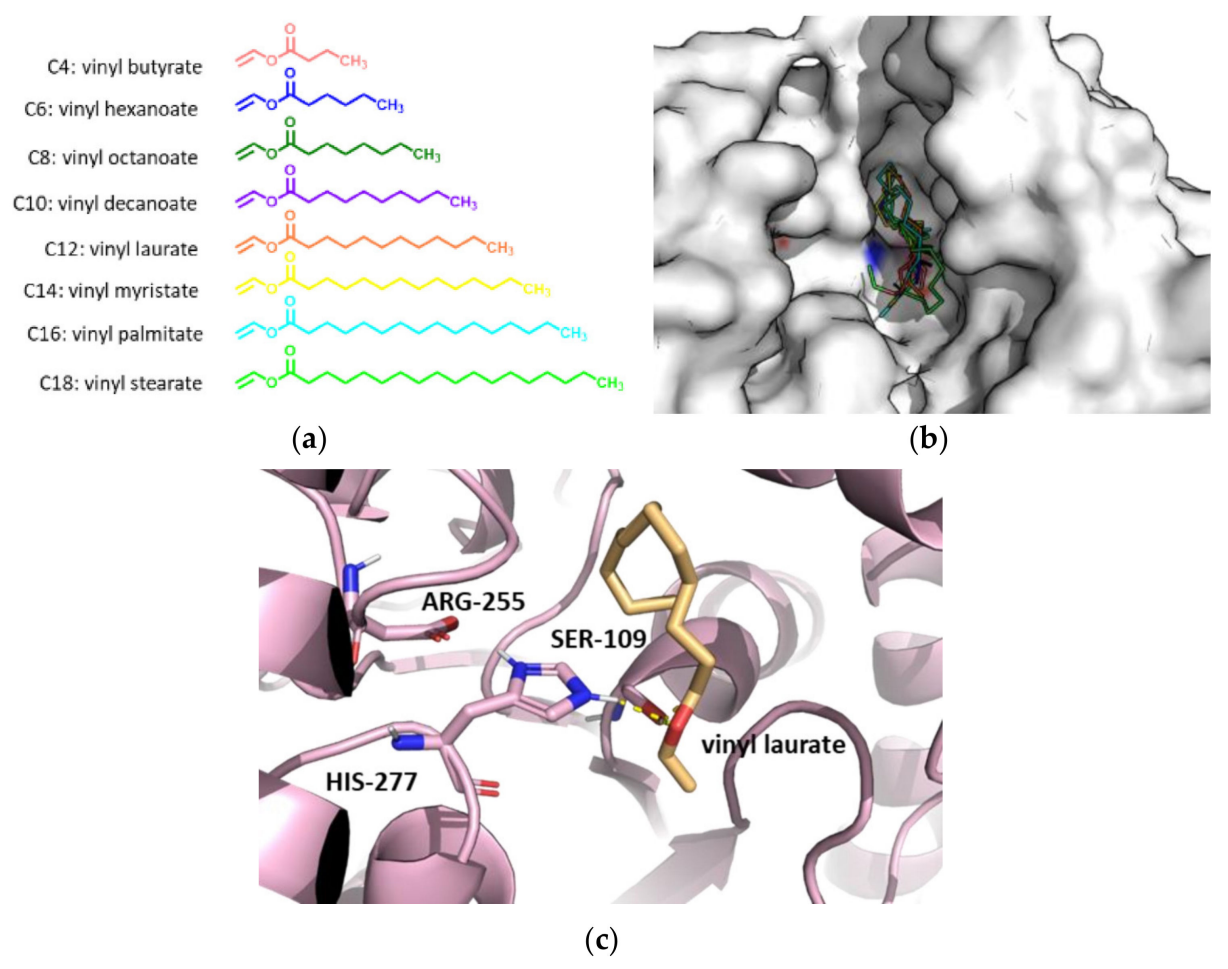
(**a**) Vinyl ester structures of different chain length; (**b**) vinyl esters docking into the cavity of PSL (protein is drawn in white surface and the ligands as coloured sticks); (**c**) vinyl laurate docking (C12) into the cavity of PSL (protein is depicted as pink cartoon, catalytic residues as pink sticks and vinyl laurate as orange sticks, and interactions are drawn as yellow dashes line).

To study the ability of the enzyme to accommodate ester chains of different lengths, we performed docking of eight vinyl esters with chains length between 4 and 18 carbons into the catalytic pocket of the previously predicted PSL model. The best fitting of the poses was chosen considering the energy of the docking and the distance to the catalytic serine (SER-109) and histidine (HIS-277). The results showed that all chains fit in the catalytic pocket with the appropriate orientation for the transesterification reaction (Figure 12).

## 3. Materials and Methods

### 3.1. Reactants and General Procedures

Lipase from *Pseudomonas stutzeri* (PSL) was purchased from Meito & Sangyo Co. Ltd.^®^, Tokyo, Japan.

All chemical reagents were obtained from Sigma-Aldrich, Munich, Germany. The lipase and L-rhamnose were dried in a desiccator under vacuum before its use.

THF was distilled before being employed. The other organic solvents and vinyl esters were dried with activated 3 Å molecular sieves before its use.

NMR spectra were recorded at 293 K on a Bruker Avance DRX 500 spectrometeroperating at 500 MHz (^1^H) or 125 MHz (^13^C). Shifts are referenced relative to deuterated solvent residual peaks (CDCl_3_). Mass spectra were recorded with an Applied Biosystems QSTAR XL. Quantitative elemental analysis by combustion of carbon, hydrogen, nitrogen and sulphur were carried out in Servicio de Microanálisis Elemental from Universidad Complutense (Madrid, Spain), using a Leco CHNS 932 Elemental Analyser. The purity of new compounds was assessed by CHNS elemental analysis, and all values were verified to be within 0.4% of the theoretical values.

Lipase activity was determined by the use of a UV-Vis-2401 Shimadzu spectrometer.

Thin layer chromatography (TLC) was carried out on aluminium sheets coated with silica gel 60 F254 (Merck, Darmstadt, Germany). TLC plates were inspected under UV light (λ = 254 nm) and developed by treatment with 10% H_2_SO_4_ in methanol, followed by heating. Column chromatography was performed with silica gel (230–400 mesh). HPLC analysis was performed using a Mediterranea Sea18 column (15 × 0.46.5 mm, Teknokroma Analítica S. A., Barcelona, Spain) and an evaporative light scattering detector (Sedex LT-ELSD-80LT).

### 3.2. Enzymatic Activity Assay

Lipase activity was measured spectrophotometrically, employing a modified assay from Palacios et al. [46] based on the hydrolysis of *p*-nitrophenylpalmitate (*p*NPP): 200 μL of a 2 mM solution of *p*NPP in 2-propanol were added to 1750 μL of 50 mM Tris-HCl buffer (pH 8) containing 0.1% of gum arabic. The mixture was pre-warmed at 37 °C and 50 μL of a lipase solution were added. The reaction was incubated at 37 °C for 2 min. Then the reaction was stopped by adding 750 μL of Marmur solution [47] (chloroform:isoamyl alcohol, 24:1). The mixture was centrifugated at 10,000 rpm for 5 min at 4 °C and the aqueous supernatant was taken off to measure the absorbance at 210 nm. The amount of released *p*-nitrophenol (*p*NP) was determined using a *p*NP calibration curve.

Control assays containing 200 μL of a 2 mM solution of *p*NPP in 2-propanol and 1800 μL of 50 mM Tris-HCl buffer (pH 8) containing 0.1% of gum arabic were also performed.

Each experimental assay was performed three times with standard deviation under 5% of the samples’ average. An activity unit was defined as the amount of enzyme necessary to hydrolyse 1 μmol of *p*NPP per minute under the conditions described above. The protein concentration was determined using Bradford method. [48] The protein calibration curve was obtained using bovine serum albumin (BSA) as standard.

### 3.3. General Procedure for the Pseudomonas stutzeri Lipase-Catalysed Synthesis of 4-O-lauroyl rhamnose (**1e**)

L-Rhamnose (**2**) (15 mg, 0.09 mmol) was dissolved in 3 mL of anhydrous THF, and the lipase from *Pseudomonas stutzeri* (3 mg, 8.2 IU/mL) and 3 Å molecular sieves (20 mg/mL) were added to the medium. The reaction was started by the addition of vinyl laurate (**3e**) (70 μL, 0.27 mmol) and the mixture was incubated at 35 °C with orbital shaking (200 rpm) until no remnant substrate could be detected by TLC and HPLC. Samples of 50 μL were withdrawn from the medium at different times and diluted in 50 μL of a mixture of acetonitrile:water, 70:30. This mixture was filtered through a 0.22 μm PTFE (polytetrafluoroethylene) syringe filter and analysed by HPLC (mobile phase: acetonitrile:water 70:30, flow: 0.7 mL/min). TLC was performed using a mixture of dichloromethane/methanol 8:1 as mobile phase.

In those experiments where the effect of the solvent was studied, **2** (15 mg, 0.09 mmol) was added to 3 mL of the solvent. The mixture was pre-warmed to 35 °C and sonicated during 10 min to favour the substrate solubility, before following the protocol described above.

### 3.4. General Procedure for the Pseudomonas stutzeri Lipase-Mediated Transesterification of 2 at a Semi-Preparative Scale

The reaction was scaled up to 30 mL: **2** (150 mg, 0.90 mmol) was dissolved in 30 mL of anhydrous THF and the lipase from *Pseudomonas stutzeri* (30 mg, 8.2 IU/mL) and 3 Å molecular sieves (20 mg/mL) were added to the medium. The reaction was started by the addition of 3 equiv. of the corresponding vinyl ester (**3a**–**3h**) and the mixture was incubated at 35 °C in an orbital shaker until no remnant substrate could be detected by TLC and HPLC. TLC and HPLC analyses were performed as described above. The mixture was filtered to remove the lipase and the molecular sieves, and the solvent was evaporated under vacuum. Products were purified through column chromatography, employing silica gel and a gradient elution with CH_2_Cl_2_:MeOH 30:1, 15:1 and 8:1.

4-*O*-lauroylrhamnose (**1e**): white solid:

α-anomer:^1^H-NMR (500 MHz, CDCl_3_) δ (ppm): 5.30 (1H, s, H-1), 4.88 (1H, t, *J* = 9.60 Hz, H-4), 4.08 (1H, dq, *J* = 9.6 and 6.1 Hz, H-5), 4.05 (1H, d, *J* = 3.0 Hz, H-2), 3.98 (1H, dd, *J* = 9.6 and 3.0 Hz, H-3), 2.5–2.3 (2H, m, H-2´), 1.7–1.5 (2H, m, H-3´), 1.41.3 (16H, m), 1.22 (3H, d, *J* = 6,1 Hz, H-6), 0.92 (t, 3H, *J* = 6.8 Hz, H-12´). ^13^C-NMR (500 MHz, CDCl_3_) δ (ppm): 175.1 (C-1´), 94.0 (C-1), 75.2 (C-4), 71.6 (C-2), 69.8 (C-3), 65.8 (C-5), 34.6 (C-2´), 32.0, 29.8, 29.7, 29.6, 29.5, 29.4, 29.3, 25.0, 22.8, 17.6 (C-6), 14.2 (C-12´).

β-anomer:^1^H-NMR (500 MHz, CDCl_3_) δ (ppm): 4.84 (1H, t, *J* = 9.60 Hz, H-4), 4.81 (1H, s, H-1), 4.05 (1H, d, *J* = 3.0 Hz, H-2), 3.67 (1H, dd, *J* = 9.6 and 3.0 Hz, H-3), 3.49 (1H, dq, *J* = 9.6 and 6.1 Hz, H-5), 2.5–2.3 (2H, m, H-2´), 1.7–1.5 (2H, m, H-3´), 1.4–1.3 (16H, m), 1.26 (3H, d, *J* = 6,1 Hz, H-6), 0.92 (t, 3H, *J* = 6.8 Hz, H-12´). ^13^C-NMR (500 MHz, CDCl_3_) δ (ppm): 174.9 (C-1´), 94.0 (C-1), 74.4 (C-4), 72.6 (C-3), 71.7 (C-2), 70.0 (C-5), 34.6 (C-2´), 32.0, 29.8, 29.7, 29.6, 29.5, 29.4, 29.3, 25.0, 22.8, 17.5 (C-6), 14.2 (C-12´).

Anal. Calc. for C_18_H_34_O_6_: C, 62.40; H, 9.89. Found: C 61.80; H, 9.86

HRMS for [M+Na]^+^ C_18_H_34_O_6_Na (*m/z*) calc. 369.23; found 369.2239.

HPLC (Acetonitrile:water 70:30, flow 0.7 mL/min): retention time 10.4 min.

Other rhamnose esters were characterised through elemental analysis and mass spectra.

4-*O*-Butyrylrhamnose (**1a**): colourless oil

Anal. Calc. for C_10_H_18_O_6_: C, 51.27; H, 7.75. Found: C, 51.12; H, 7.72

HRMS for [M+Na]^+^ C_10_H_18_O_6_Na (*m/z*) calc. 257.09; found 257.0991.

HPLC (Acetonitrile:water 70:30, flow 0.7 mL/min): retention time 2.7 min.

4-*O*-Hexanoylrhamnose (**1b**): white solid

Anal. Calc. for C_12_H_22_O_6_: C, 54.95; H, 8.45. Found: C, 54.79; H,8.41

HRMS for [M+Na]^+^ C_12_H_22_O_6_Na (*m/z*) calc. 285.12; found 285.1299.

HPLC (Acetonitrile:water 70:30, flow 0.7 mL/min): retention time 3.2 min.

4-*O*-Octanoylrhamnose (**1c**): white solid

Anal. Calc. for C_14_H_26_O_6_: C, 57.91; H, 9.03. Found: C, 57.82; H,9.01

HRMS for [M+Na]^+^ C_14_H_26_O_6_Na (*m/z*) calc. 313.16; found 313.1613.

HPLC (Acetonitrile:water 70:30, flow 0.7 mL/min): retention time 4.1 min.

4-*O*-Decanoyl rhamnose (**1d**): white solid

Anal. Calc. for C_16_H_30_O_6_: C, 60.35; H, 9.50. Found: C, 60.20; H, 9.47

HRMS for [M+Na]^+^ C_16_H_30_O_6_Na (*m/z*) calc. 341.19; found 341.1926.

HPLC (Acetonitrile:water 70:30, flow 0.7 mL/min): retention time 6.2 min.

4-*O*-Myristylrhamnose (**1f**): white solid

Anal. Calc. for C_20_H_38_O_6_: C, 64.14; H, 10.23. Found: C, 63.97; H, 10.19

HRMS for [M+Na]^+^ C_20_H_38_O_6_Na (*m/z*) calc. 397.25; found 397.2552.

HPLC (Acetonitrile:water 80:10, flow 0.7 mL/min): retention time 8.5 min.

4-*O*-Palmitoylrhamnose (**1g**): white solid

Anal. Calc. for C_22_H_42_O_6_: C, 65.64; H, 10.52. Found: C, 65.42; H, 10.51

HRMS for [M+Na]^+^ C_22_H_42_O_6_Na (*m/z*) calc. 425.28; found 425.2864.

HPLC (Acetonitrile:water 90:10, flow 0.7 mL/min): retention time 13.6 min.

4-*O*-Stearyl rhamnose (**1h**) white solid

Anal. Calc. for C_24_H_46_O_6_: C, 66.94; H, 10.77. Found: C, 66.75; H, 10.74

HRMS for [M+Na]^+^ C_24_H_46_O_6_Na (*m/z*) calc. 453.31; found 453.3174.

HPLC (Acetonitrile:water 90:10, flow 0.7 mL/min): retention time 23.5 min.

### 3.5. Computational Methods

#### 3.5.1. Pseudomonas stutzeri Lipase Protein Modelling

Homology modelling for PS Lipase (WP_133457103.1) was carried out using the I-Tasser [49,50,51] (http://zhanglab.ccmb.med.umich.edu/I-TASSER/, accessed on 26 June 2021) and Swiss-Model [52] (https://swissmodel.expasy.org/, accessed on 7 February 2022) protein prediction servers. Based on the X-ray structure of *P. aeruginosa* lipase (PDB ID: 1ex9) [53], which was selected as template (sequence identity 79.79% over 100% of sequence), because an X-ray structure of the more homologue lipase from *P. mendocina* was not available. Both servers confirmed the same homology model, which was used to perform the MD simulations. The structure setup entailed adding hydrogen atoms, to assign atom types and charges according to AMBER ff14SB force field [54], and to determine the protonation state of titratable residues at pH 7 using H++ web server, version 3.0 [55,56,57]. The Poisson-Boltzmann (PB) method [58] was used, at pH 7, 0.15 M salt concentration, with internal and external dielectric constants of 4 and 80. In the structures, the catalytic serine (SER-109) [43] was esterified using the lauric acid moiety. The resultant enzyme was further minimised with the same force field.

#### 3.5.2. Ligand Preparation

Three-dimensional structures for α- and β-rhamnose were built using glycam carbohydrate builder web server (https://glycam.org/, accessed on 7 February 2022), and 3D structures of vinyl esters were built with the interactive molecular graphics programme PyMOL [59], which was also employed for structure visualisation and molecular editing. The ground state geometry of the ligands were optimised and fitted to the atoms as AMBER atom types and RESP charges using the programme antechamber (AmberTools16 [45], URL: ambermd.org, San Francisco, CA, USA).

#### 3.5.3. Docking

Local docking of the studied compounds into the active site of PS lipase was done by using AutoDock 4.2 [44]. Vinyl esters were docked in a well-defined cavity where the catalytic triade are located (residues SER-109, ARG-255 and HIS-277). The default Lamarckian genetic algorithm parameters and a grid of 60 × 40 × 60 points (spacing 0.3675 Å) were chosen. The most favourable poses were selected according to its predicted docking energy and the distances between SER-109 and vinyl esters. α- and β-rhamnose were docked in the catalytic pocket with SER-109 already esterified, once again Lamarckian genetic algorithm parameters were chosen and a grid of 35 × 35 × 35 points (spacing 0.3675 Å). The mechanism of the lipase and the distance to the catalytic triad were taken as references to select the poses. Two complexes were selected for PS lipase, one with α-rhamnose and another with β-rhamnose; in both complexes, the distance between the hydroxyl group at position 4 of the sugar and SER-109 was no more than 2.8 Å. The resultant complexes were used as the starting point for molecular dynamics studies.

#### 3.5.4. Molecular Dynamics Simulation in THF as Solvent

The complexes were immersed in cubic boxes of THF large enough to guarantee that the shortest distance between the solute and the edge of the box was greater than 15 Å. The solvent was represented explicitly at the molecular level of THF molecules. As THF is not a standard solvent in the AMBER force field, it was necessary to first determine the parameters of the THF to be able to perform the simulations. We have used xleap programme (AmberTools16 [45], URL: ambermd.org, accessed on 26 June 2021) to draw the molecule and obtain the structure in pdb format. The ground state geometry of THF solvent molecule was optimised and fitted to the atoms as AMBER atom types and RESP charges using the programme antechamber (AmberTools16 [45]). Parmchk programme (AmberTools16 [45,60]) was used to create force field modification (frcmod) files that fill in missing parameters. Finally, to pack the protein inside the solvents box we have used the standard protocol of Packmol programme [7,61]. Packmol created an initial point for the molecular dynamics simulations of the complexes, and then the MD simulation protocol was carried out using AMBER16 package [45]. The starting structures, prepared as indicated above, were simulated in the NPT ensemble with the periodic boundary conditions and particle mesh Ewald method to treat long-range electrostatic effects [62]. The protocol was as follows: Three consecutive minimisations were performed: (i) involving only hydrogen atoms, (ii) involving only the water molecules and ions, and (iii) involving the entire system. The system was then heated and equilibrated in two steps: (i) 20 ps of MD heating the whole system from 100 to 300 K and (ii) equilibration of the entire system during 100 ps at 300 K. The equilibrated structure was the starting point for 50 ns of MD simulations carried out using the pmemd_cuda.SPFP at constant temperature (300 K), pressure (1 atm) and the standard ff14SB force field parameters. The constraint algorithm SHAKE [63] was used to keep bonds involving H atoms at their equilibrium length, allowing a 2 fs time step for the integration of Newton’s equations of motion. The *cpptraj* module [64] in AMBER16 was employed for data processing and geometry analysis of the calculated trajectories.

#### 3.5.5. Analysis of MD Trajectories

The stability of the complex was evaluated by calculating the root-mean-square-deviation (RMSD) of the Cα atoms along the trajectories, using their starting structures as reference.

## 4. Conclusions

In summary, the results described above are very promising when compared with similar processes reported in the literature regarding the enzymatic synthesis of rhamnose fatty acid esters. PSL is a very convenient lipase for the synthesis of rhamnose 4-*O*-monoesters in polar solvents, such as THF or acetone, using vinyl fatty acid esters as acyl donors. In addition, the reaction can be performed in more a sustainable solvent, such as Me-THF, with high conversion and keeping total regioselectivity.

To our knowledge, this is the first report of an enzymatic method to obtain monoacylated rhamnose with short, medium and long fatty acid chains in one step, with a very high yield and total regioselectivity toward the C-4 position.

The influence of different reaction conditions, such as temperature, a substrates molar ratio, organic reaction medium and acyl donor chain-length, was studied. Thus, we could demonstrate that the lipase PSL efficiently catalyses the transesterification reaction of rhamnose with fatty acid vinyl esters in the presence of polar solvents, allowing high conversion and regioselectivity. Furthermore, rhamnose esters are one of the most studied biosurfactants and present multiple applications in food, cosmetic, detergent and pharmaceutical industries [22,23,24,26,27,28]. They are currently prepared by industrial fermentation, using low-efficiency and costly downstream processes [28,29]. The results reported in the present work provide two major advantages: first, a lipase that catalyses the transesterification reaction with high regioselectivity toward the C-4 position of rhamnose and, secondly, we performed this regioselective acylation under environmental friendly conditions, by using green solvents suitable to achieve high conversion (86%) and avoiding the use of any toxic reagent and solvents required in the classical chemical process.

In addition, the mechanism of the transesterification reaction of rhamnose and vinyl esters with high regioselectivity by PSL was analysed. By using the homology model of this lipase and carried out docking studies and molecular dynamic simulations, we have demonstrated that this enzyme is highly selective for the C-4 position. Computational studies were used to obtain the models of interaction between rhamnose and acetylated lipase, revealing that, in addition to interactions with the catalytic triad, hydrogen bonding and van der Waals interactions that fix the position of the sugar in the active site are crucial for C-4 regioselectivity. The results reasonably explain the high regioselectivity of PSL in the transesterification reaction of rhamnose and vinyl laurate. The analysis of molecular dynamics simulations on the docking complexes reinforces the conclusions about regioselectivity of PSL toward the C-4 position of rhamnose.

These studies also explained the ability of this lipase to accommodate ester chains of different lengths. Small, medium or large fatty acid chains are able to enter to the active site of the enzyme to react with the Ser-109, which can be explain by the large hydrophobic pocket observed in the active side of the enzyme.

These results may contribute to the design and engineering of other lipases, especially those with improved regioselectivity for sugar fatty acid esters.

## Figures and Tables

**Figure 6:**
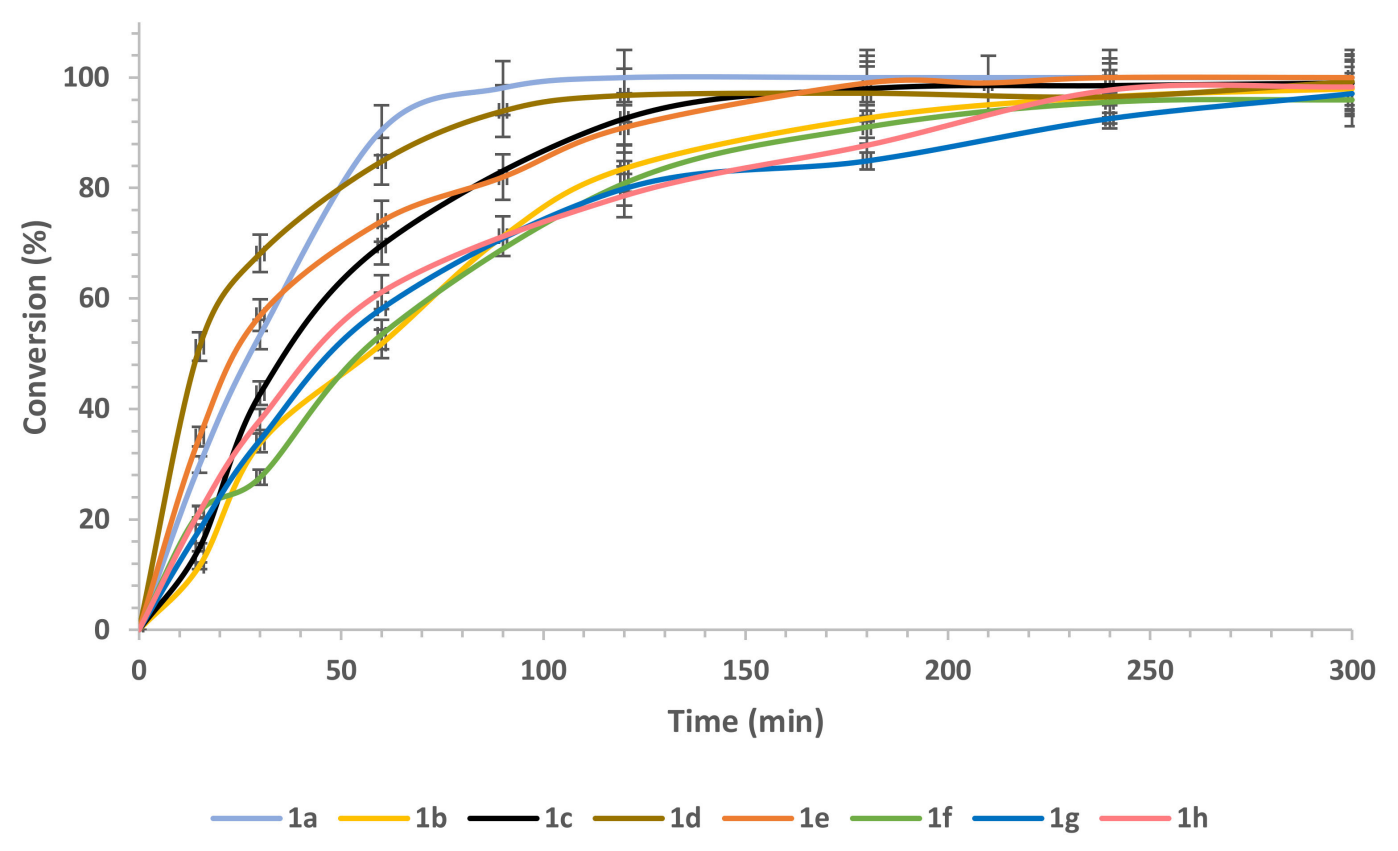
PSL catalysed transesterification reaction of **2** employing acyl donors with different chain length.

## Data Availability

Not applicable.

## Supplementary Materials

Supplementary Materials are available online at https://www.mdpi.com/article/10.3390/ijms23042239/s1.
