# Supplementary material for: Enzymatic Synthesis and Molecular Modelling Studies of Rhamnose Esters Using Lipase from Pseudomonas stutzeri
Source: Int J Mol Sci. 2022 Feb 17;23(4):2239. doi: 10.3390/ijms23042239 (PMC8876684; doi:10.3390/ijms23042239)
Supplement: Supplementary file 1 [file ijms-23-02239-s001.zip › ijms-1574082-supplementary.pdf]

Supplementary Material for

Enzymatic Synthesis and Molecular Modeling Studies of Rhamnose Esters using Lipase from *Pseudomonas stuzeri*

Cecilia Garcia-Oliva [1], Almudena Perona [1], Ángel Rumbero [2], Pilar Hoyos [1], and María J. Hernáiz [1,*]

[1] Department of Chemistry in Pharmaceutical Sciences, Faculty of Pharmacy, Complutense University of Madrid, Plaza Ramón y Cajal, E 28040 Madrid, Spain

[2] Department of Organic Chemistry, Autonomous University of Madrid, Cantoblanco, 28049 Madrid, Spain

* Correspondence: mjhernai@ucm.es

a)

b)

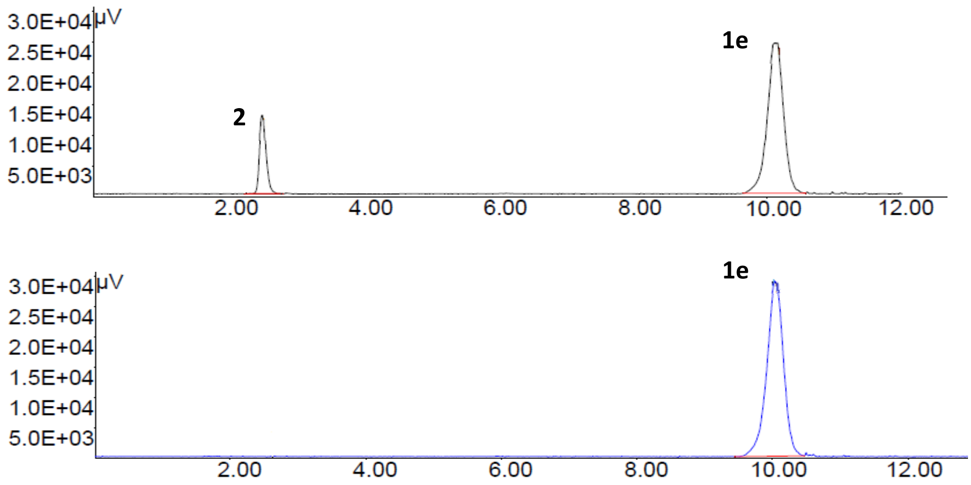

Figure S1. HPLC analyses of different reaction times of the PSL-catalysed transesterification reaction of **2** with the acyl donor **3e** (synthesis of **1e**); mobile phase: acetonitrile:water, 70:30, flow 0.7 mL/min: a) reaction time 2h; b) reaction time 3 h.

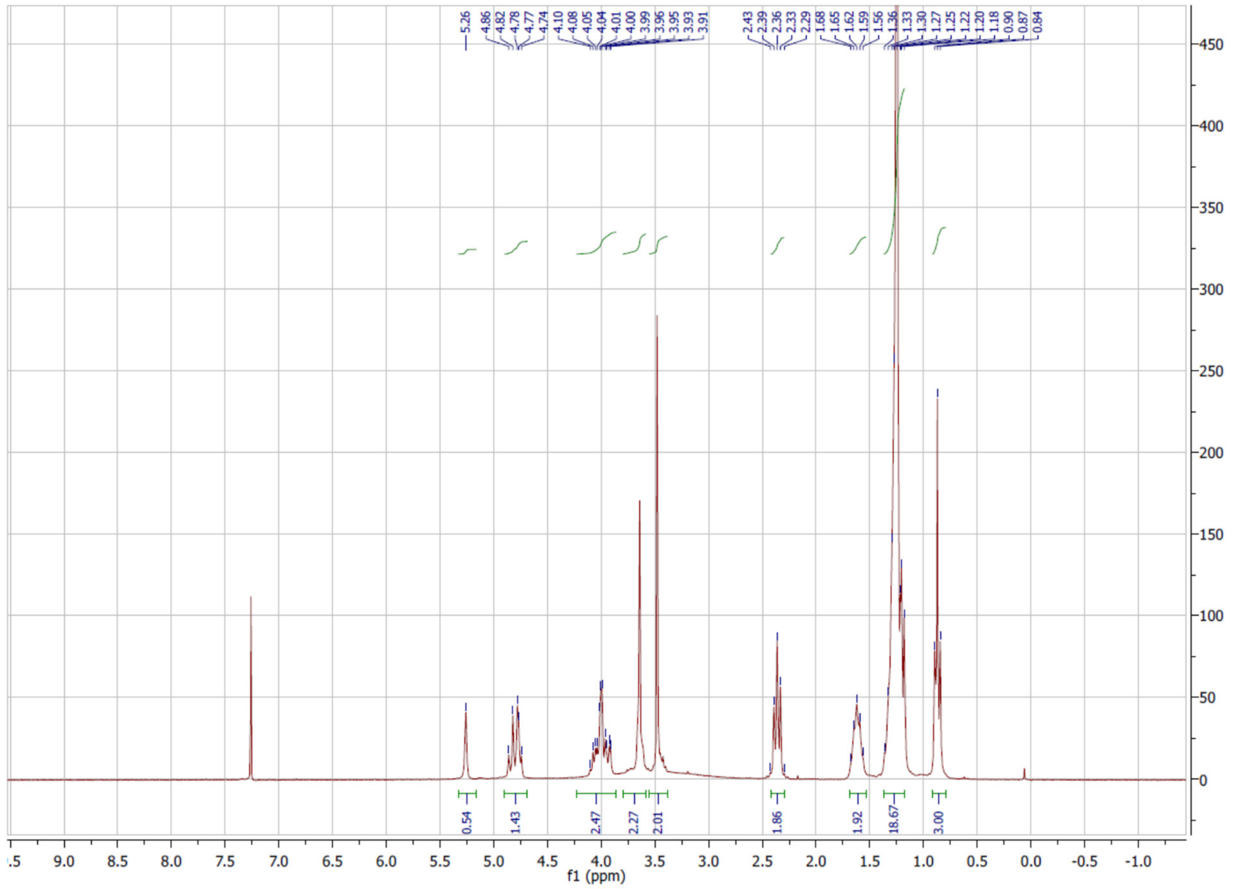

Figure S2. ^{1}H-NMR spectra 4-*O*-lauroylrhamnose (**1e**).

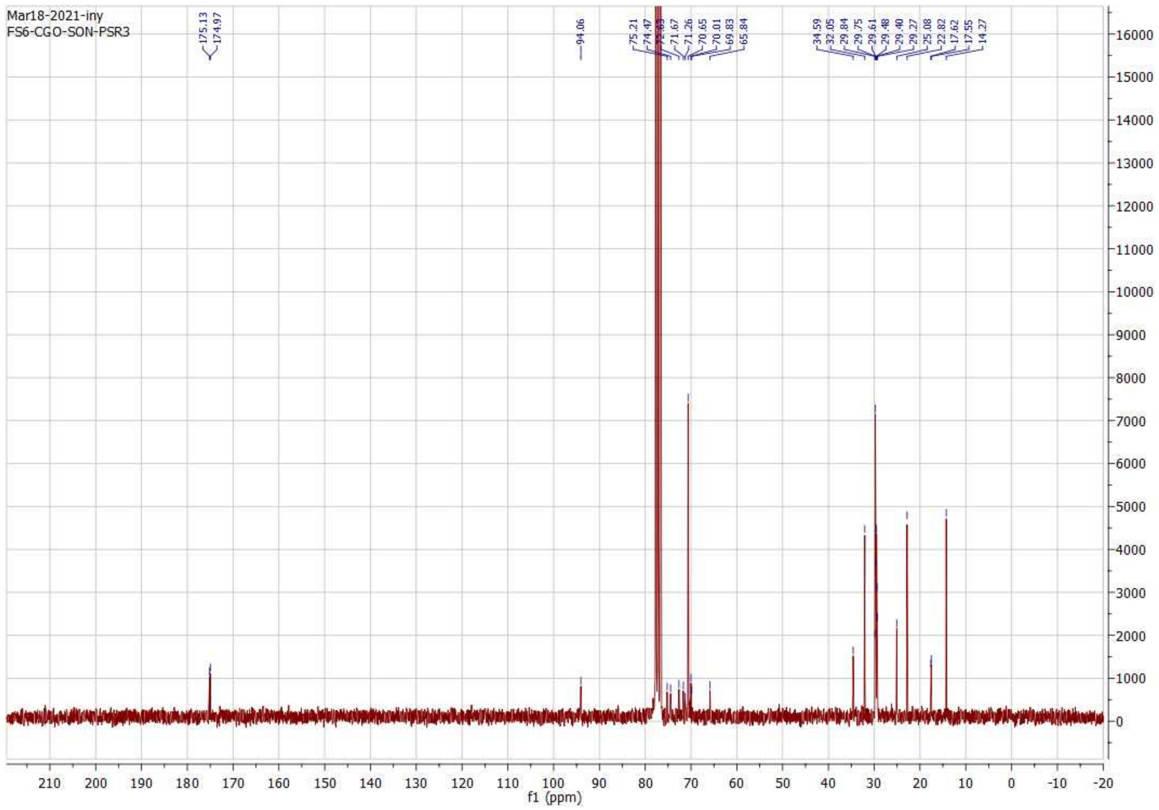

Figure S3. ^{13}C-NMR spectra 4-O-lauroylrhamnose (**1e**).

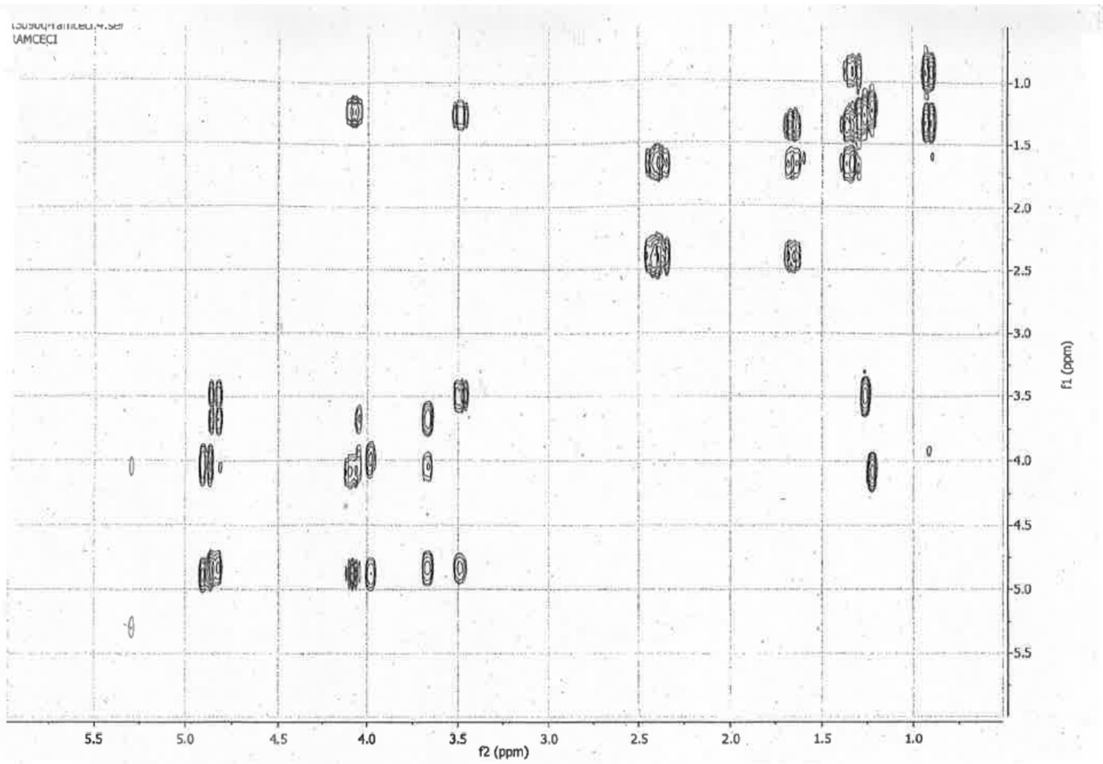

Figure S4. COSY spectrum of 4-O-lauroylrhamnose (**1e**)

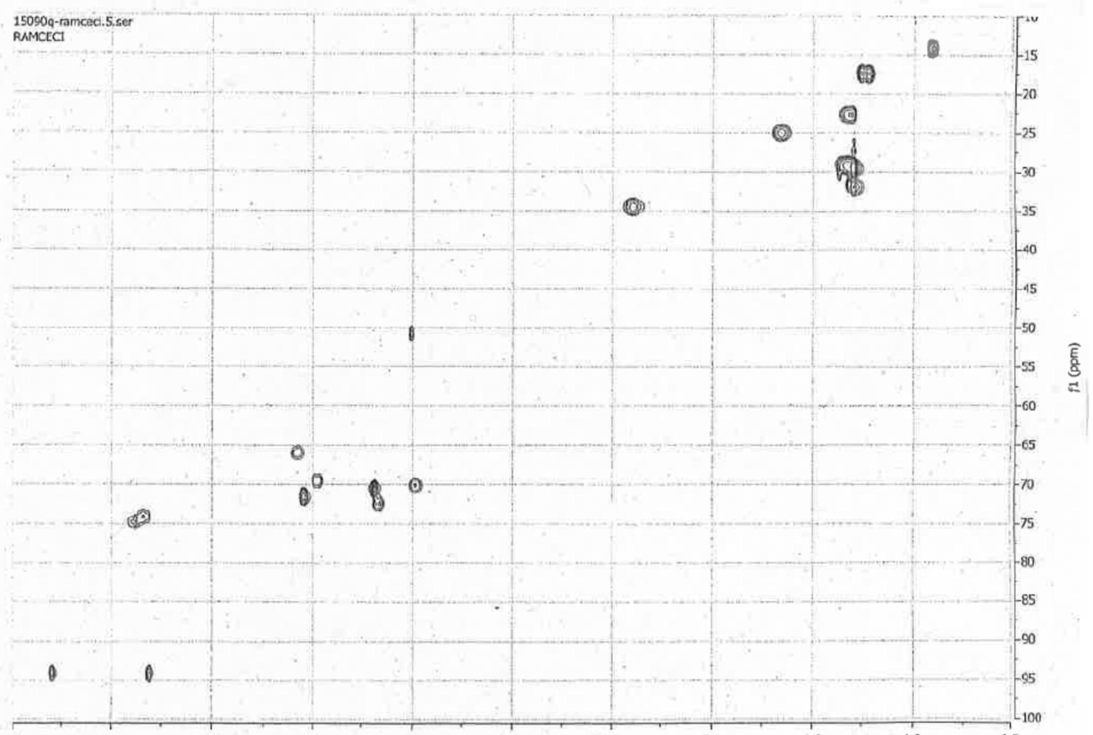

Figure S5. HSQC spectrum of 4-O-lauroylrhamnose (**1e**)

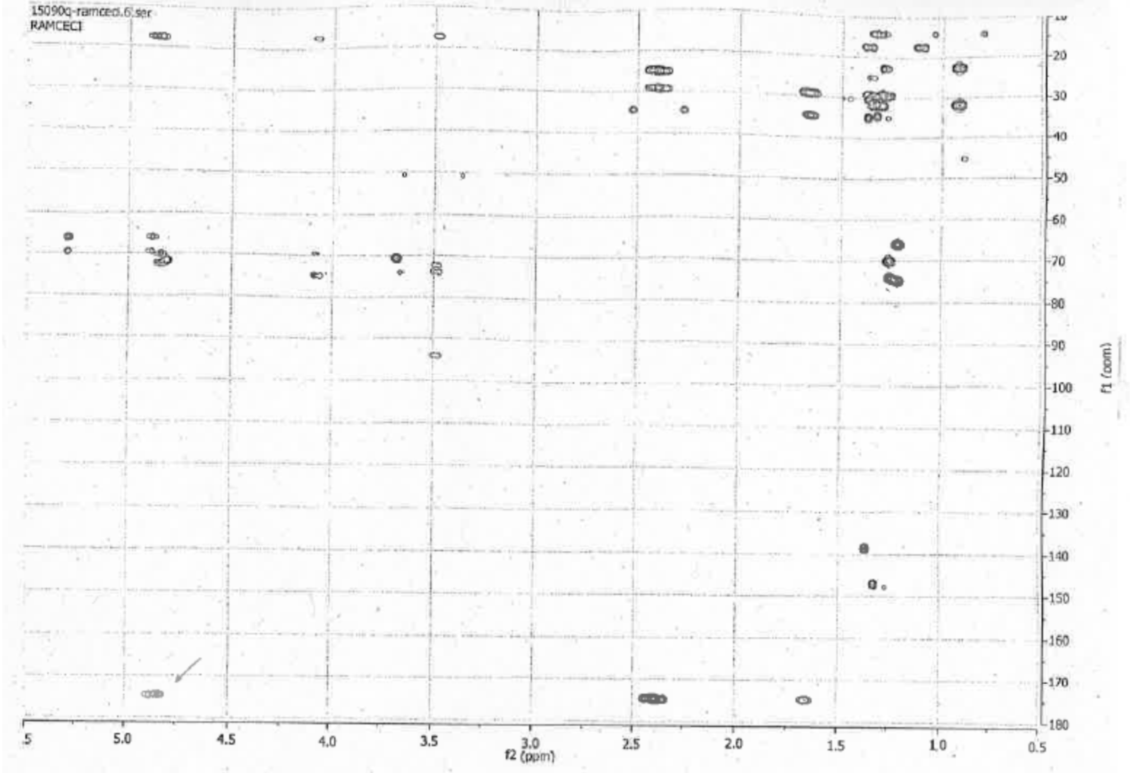

Figure S6. HMBC spectrum of 4-O-lauroylrhamnose (**1e**)

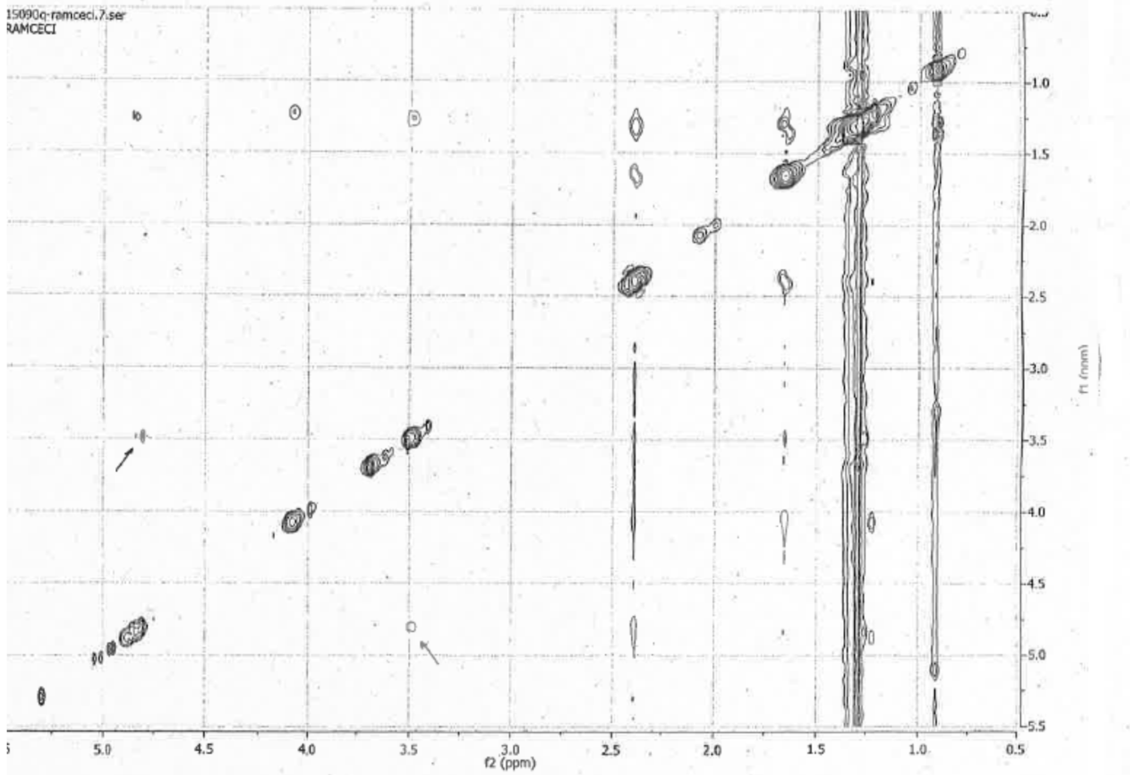

Figure S7. ROESY spectrum of 4-O-lauroylrhamnose (**1e**)

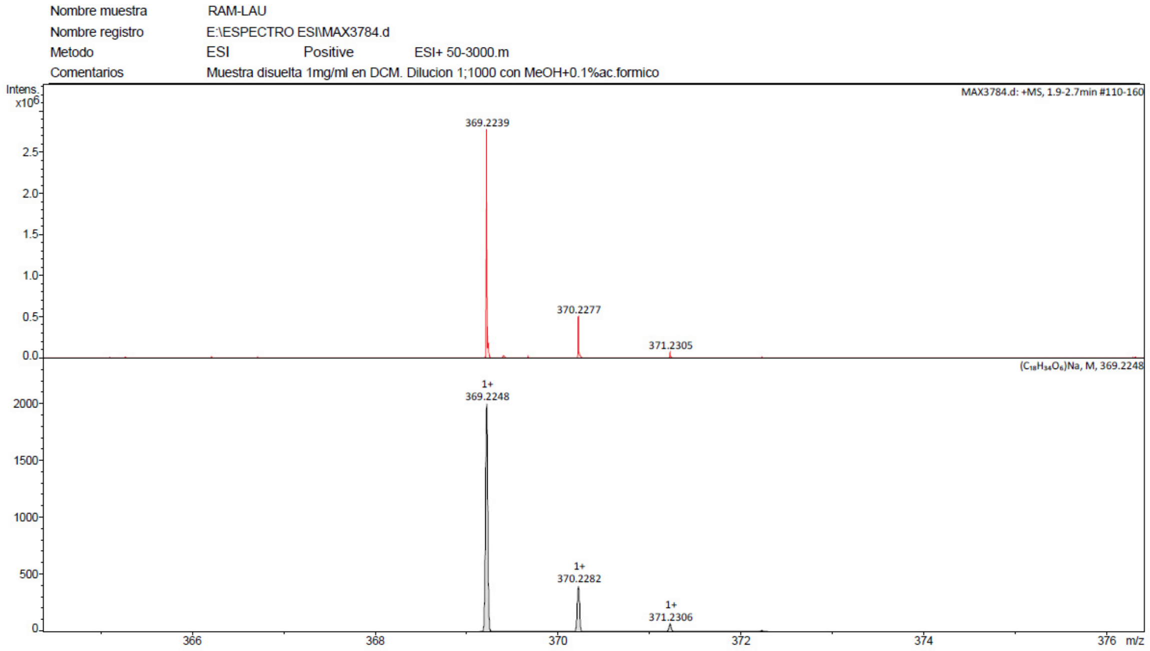

Figure S8. MS spectra for 4-*O*-lauroylrhamnose (**1e**).

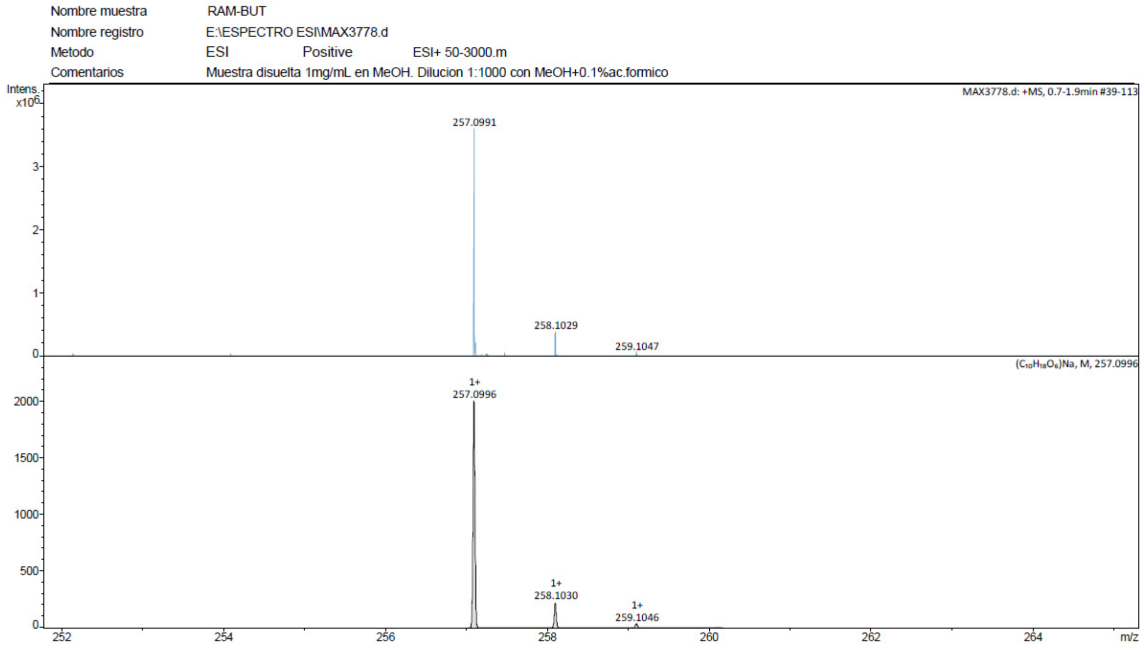

Figure S9. MS spectra for 4-*O*-butyrylrhamnose (**1a**).

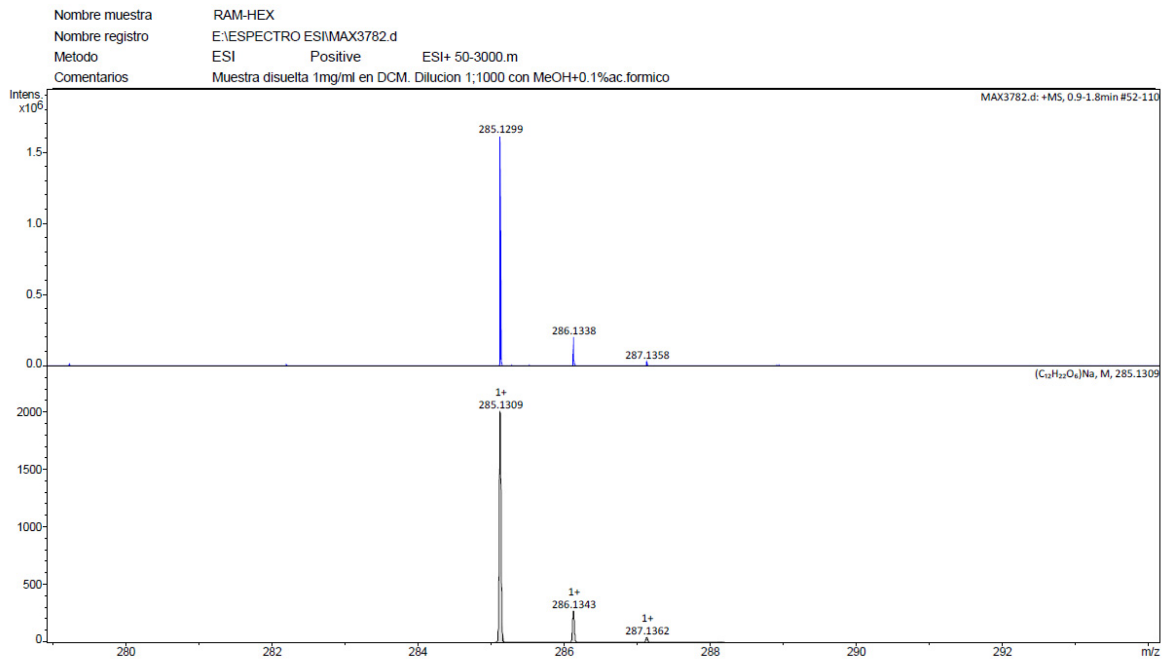

Figure S10. MS spectra for 4-*O*-hexanoylrhamnose (**1b**).

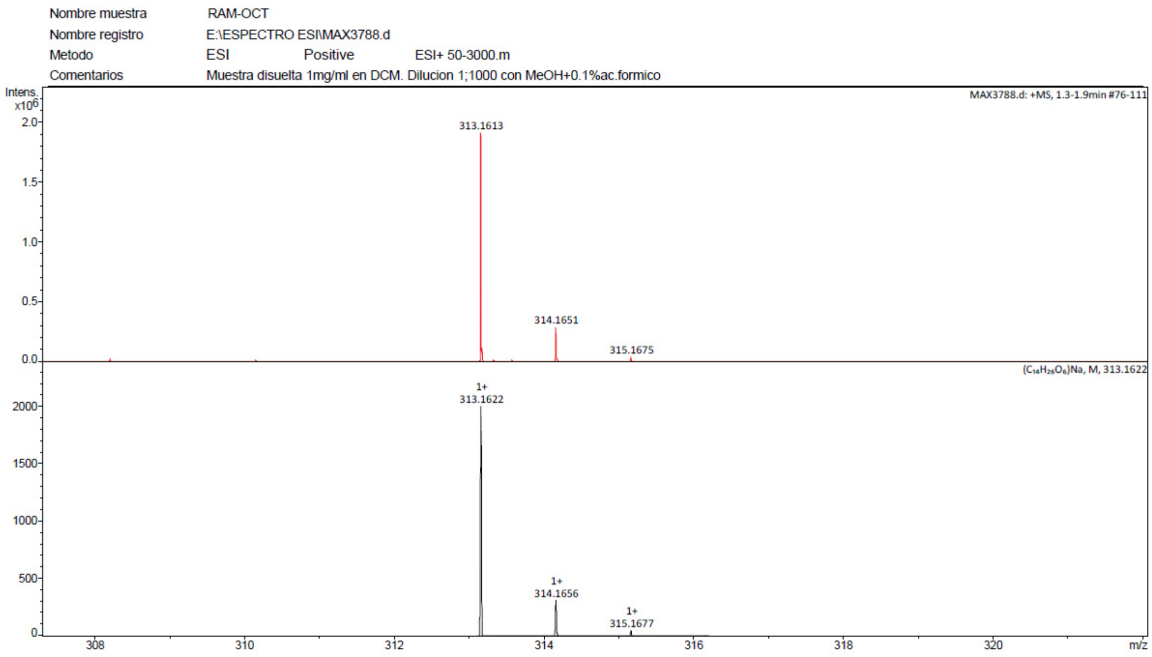

Figure S11. MS spectra for 4-*O*-octanoylrhamnose (**1c**).

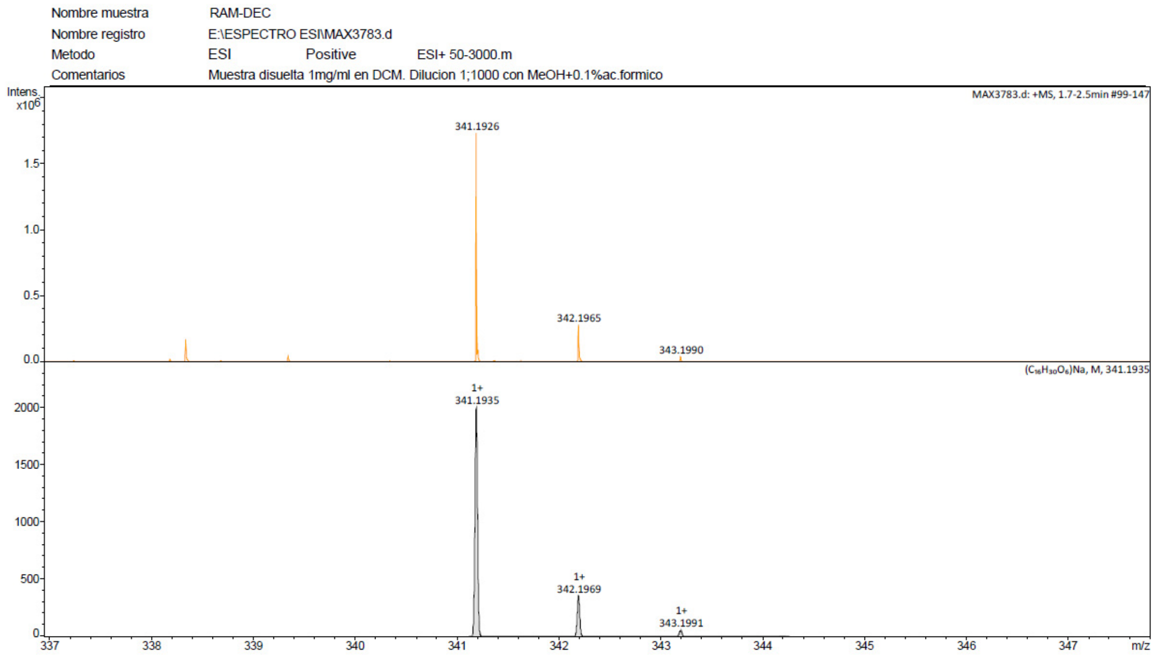

Figure S12. MS spectra for 4-*O*-decanoylrhamnose (**1d**).

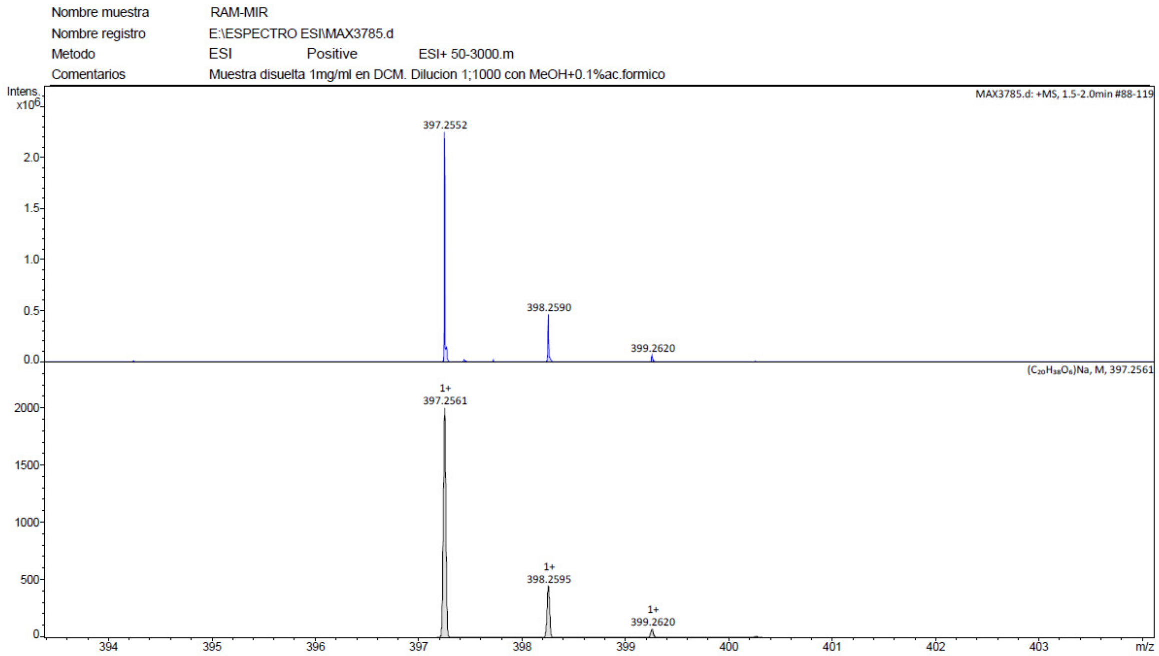

Figure S13. MS spectra for 4-*O*-myristylrhamnose (**1f**).

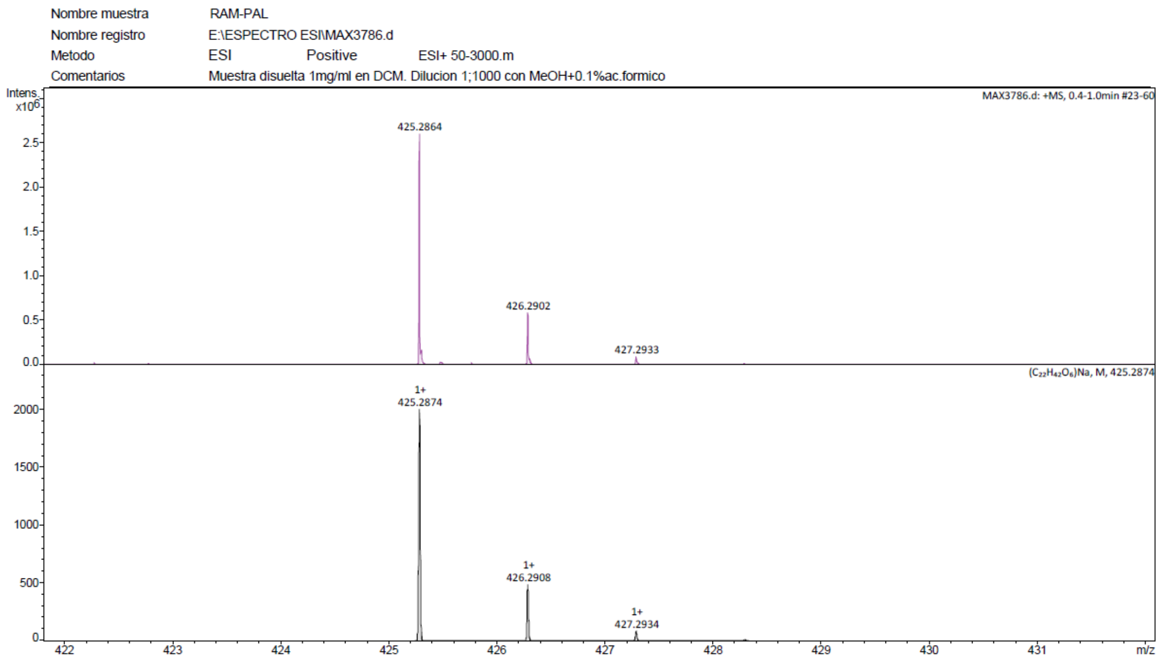

Figure S14. MS spectra for 4-*O*-palmitoylrhamnose (**1g**).

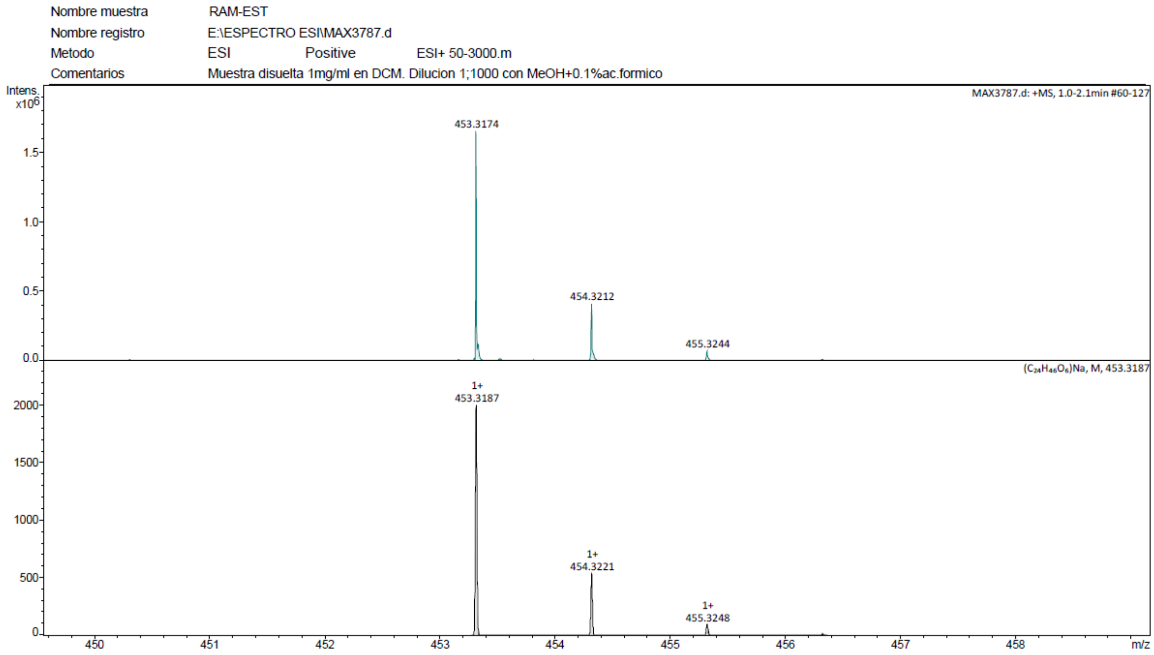

Figure S15. MS spectra for 4-*O*-stearylrhamnose (**1h**).
